# When Long-Range Zero-Lag Synchronization is Feasible in Cortical Networks

**DOI:** 10.3389/fncom.2012.00049

**Published:** 2012-07-27

**Authors:** Atthaphon Viriyopase, Ingo Bojak, Magteld Zeitler, Stan Gielen

**Affiliations:** ^1^Donders Institute for Brain, Cognition and Behavior, Radboud University Nijmegen (Medical Centre)Nijmegen, Netherlands; ^2^School of Psychology, Centre for Computational Neuroscience and Cognitive Robotics, University of BirminghamBirmingham, UK

**Keywords:** long-range synchronization, spike-timing dependent plasticity, zero-lag synchronization

## Abstract

Many studies have reported long-range synchronization of neuronal activity between brain areas, in particular in the beta and gamma bands with frequencies in the range of 14–30 and 40–80 Hz, respectively. Several studies have reported synchrony with zero phase lag, which is remarkable considering the synaptic and conduction delays inherent in the connections between distant brain areas. This result has led to many speculations about the possible functional role of zero-lag synchrony, such as for neuronal communication, attention, memory, and feature binding. However, recent studies using recordings of single-unit activity and local field potentials report that neuronal synchronization may occur with non-zero phase lags. This raises the questions whether zero-lag synchrony can occur in the brain and, if so, under which conditions. We used analytical methods and computer simulations to investigate which connectivity between neuronal populations allows or prohibits zero-lag synchrony. We did so for a model where two oscillators interact via a relay oscillator. Analytical results and computer simulations were obtained for both type I Mirollo–Strogatz neurons and type II Hodgkin–Huxley neurons. We have investigated the dynamics of the model for various types of synaptic coupling and importantly considered the potential impact of Spike-Timing Dependent Plasticity (STDP) and its learning window. We confirm previous results that zero-lag synchrony can be achieved in this configuration. This is much easier to achieve with Hodgkin–Huxley neurons, which have a biphasic phase response curve, than for type I neurons. STDP facilitates zero-lag synchrony as it adjusts the synaptic strengths such that zero-lag synchrony is feasible for a much larger range of parameters than without STDP.

## Introduction

Coupling between different oscillators and pacemakers can generate a large range of different behaviors and has been a topic of study in many different conditions, for example in cardiac pacemaking and chemical oscillations (see e.g., Goldbeter, 1996; Koch and Segev, 1998; Roxin et al., 2005). A special case is the interaction between neurons, which gives rise to neuronal oscillations in particular frequency bands. Neuronal gamma band synchronization has been reported in many species and in a large number of brain structures for a variety of sensory and motor tasks (Gray et al., 1989; Fries et al., 2001, 2008; Pesaran et al., 2002; Schoffelen et al., 2005). This large-scale synchronization of multiple cortical areas has been postulated as a potential mechanism for integration and coordination of neuronal activity in cognitive tasks (Engel et al., 1992; Singer and Gray, 1995; Fries, 2005).

The first studies on this topic presented experimental evidence that the relative phase of gamma oscillations in widely separated brain areas is near zero (Frien et al., 1994; Roelfsema et al., 1997; Castelo-Branco et al., 1998; Rodriguez et al., 1999; Gross et al., 2004). This result was remarkable since synchronization requires interactions between distant brain areas, which come with considerable delays due to axonal conduction and synaptic transmission. Many further studies hence investigated how distant oscillatory brain regions can synchronize at zero-lag in spite of non-negligible delays. Several theoretical studies have argued that direct mutual pulse-coupling between two dynamical systems with delays and excitatory synapses cannot easily lead to zero-lag synchrony (Ernst et al., 1995, 1998; Goel and Ermentrout, 2002; Zeitler et al., 2009). Therefore, Fischer et al. (2006) and Vicente et al. (2008) suggested that zero-lag synchronization between brain areas might be mediated by a third (relay) oscillator. A potential candidate for this neuronal relay oscillator is the thalamus (Gollo et al., 2010; Theyel et al., 2010).

In order to obtain a better understanding of the possibilities for zero-lag synchronization of distant brain areas, we have investigated the proposed network of neuronal oscillators coupled indirectly by a relay oscillator (Fischer et al., 2006; Vicente et al., 2008). We have investigated the model with both type I Mirollo–Strogatz neurons, as well as with type II Hodgkin–Huxley neurons. We focused in particular on the robustness of zero phase synchronization as a function of both the delay time between oscillators and the strength of synaptic coupling. Various types of synaptic coupling were investigated, including Spike-Timing Dependent Plasticity (STDP), since STDP has been suggested to contribute to efficient information transmission (Buonomano and Maass, 2009; Lindner et al., 2009; Hennequin et al., 2010). Our results show that zero-lag synchrony can occur, especially for models with Hodgkin–Huxley type II neurons. STDP facilitates zero-lag synchrony as STDP modifies synaptic strengths and thereby allows a larger range of initial synaptic strengths that may lead at zero-lag synchronization.

## Materials and Methods

**Figure 1:**
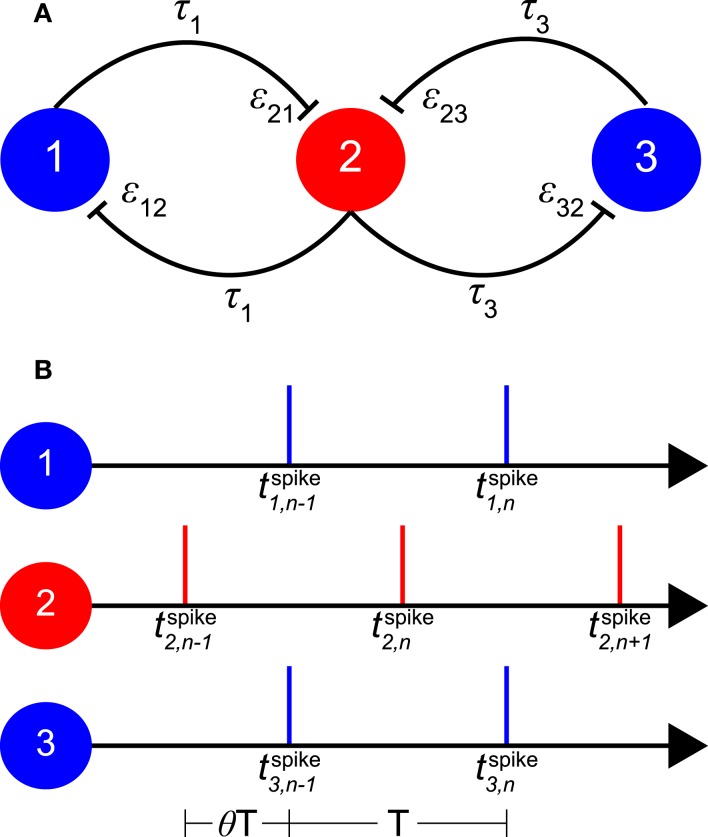
**Schematic of the model and its 1:1 phase-locked zero-lag synchrony mode**. **(A)** Sketch of oscillators 1 and 3 with bi-directional pulse-coupling via relay oscillator 2. ε*_ij_* represents the excitatory synaptic weight from oscillator *j* to oscillator *i*, τ*_k_* the conduction delay between relay oscillator 2 and oscillator *k*. **(B)** An example of 1:1 phase-locked zero-lag synchrony mode. Vertical bars represent the spike times of the oscillators. *T*_0_ is the intrinsic period of the oscillators, *T* < *T*_0_ the common period of the oscillators in the phase-locked mode, and θ*T* the delay between the firing of the relay and the outer oscillators. $t_{1,n}^{\text{spike}},$ $t_{2,n}^{\text{spike}},$ and $t_{3,n}^{\text{spike}}$ are the *n*-th spike times of oscillators 1, 2, and 3, respectively.

The model consists of three coupled neuronal oscillators (see Figure 1A). Each oscillator can be considered as a single neuron or as a population of neurons, where the activity of the neurons within each population is assumed to be homogeneous and highly synchronized. As illustrated in Figure 1A, the “relay” or “inner” oscillator (oscillator 2) is coupled bi-directionally with two “outer” oscillators (oscillators 1 and 3). The outer oscillators are only coupled with the relay oscillator but not directly to each other. As starting point we assume that all three oscillators are identical with the same intrinsic firing period *T*_0_.

We have used the Mirollo–Strogatz (MS) phase oscillator (Mirollo and Strogatz, 1990) and the classical Hodgkin and Huxley (HH) neuron (Hodgkin and Huxley, 1952) with parameters given in Vicente et al. (2008). For the MS phase oscillator, *T*_0_ is chosen to be 25 ms and *T*_0_ of the HH neuron is 14.66 ms with the parameters given in Vicente et al. (2008). In Figure 1A ε*_ij_* represents the coupling strength from pre-synaptic oscillator *j* to postsynaptic oscillator *i*. All synaptic couplings in the model are excitatory. The delay time τ*_k_* represents the conduction time for spikes along axons that connect oscillator *k* with the relay oscillator. Here we make the simplifying assumption that delay times are constant and symmetric (i.e., the delay time from oscillator *i* to *j* is equal to that from oscillator *j* to *i*). These delay times are typically considerably shorter than the period of neuronal oscillations (Fries, 2005). Therefore in the following we will only consider conduction delays shorter than *T*_0_/2, with τ*_k_* expressed as a fraction of the intrinsic period *T*_0_ and thus in the range between 0 and 0.5.

The MS phase oscillator (Mirollo and Strogatz, 1990) is characterized by a voltage-like state variable *f* ∈ [0, 1], which increases monotonically from 0 toward the threshold value *f* = 1. Within a cycle, the state of the uncoupled neuronal oscillator is defined by a monotonically increasing concave function *f *(φ):[0, 1] → [0, 1]:

$$\begin{array}{llll} f\left(\varphi \right) & =\frac{1}{b}\ln\left[1+\left(e^{b}-1\right)\varphi \right], & & \text{(1)}\\ \frac{d\varphi }{dt} & =\frac{1}{T_{\text{0}}}, & & \text{(2)} \end{array}$$

with a phase variable φ ∈ [0, 1] and a dissipation parameter *b*. *T*_0_ is the intrinsic firing period of the oscillator. When the threshold is reached, the oscillator fires, the state variable *f* is reset to zero, and the cycle repeats. As in Ernst et al. (1995) and Zeitler et al. (2009), the setting *b* = 3 is used throughout this study. The MS neuron is a so-called type I neuron (Izhikevich, 2007), where excitatory input always gives a phase advance of the neuronal oscillator.

For the classical HH neuron the membrane potential *V* is governed by the differential equation:

$$\begin{array}{llll} C\frac{dV}{dt} & =-g_{\text{Na}}m^{3}h\left(V-E_{\text{Na}}\right)-g_{\text{K}}n^{4}\left(V-E_{\text{K}}\right)-g_{\text{L}}\left(V-E_{\text{L}}\right) & & \\ & \;+I_{\text{ext}}+I_{\text{syn}}, & & \text{(3)} \end{array}$$

with the membrane capacitance *C* and the maximal conductances of sodium *g*_Na_, potassium *g*_K_, and leakage *g*_L_. The corresponding reversal potentials (*E*_Na_, *E*_K_, *E*_L_) and the external current *I*_ext_ are as given in Vicente et al. (2008). The voltage-gated ion channels m, h, and n are described by first order differential equations. Note that the expression for α*_n_* in Vicente et al. (2008) was not correct and should read α*_n_*(*V*) = [(*V* + 55)/100]/{1 − exp[ − 0.1(*V* + 55)]}. The excitatory synaptic current *I*_syn_ is −ε*_ij_S*(*t*)*V*, where ε*_ij_* is the maximum synaptic conductance and *S*(*t*) is the Dirac delta function (in case of an “instantaneous synapse”) or an alpha function (in case of an “alpha synapse”). This classical HH neuron is a so-called type II neuron (Izhikevich, 2007), where excitatory input in early phases of the firing cycle causes a phase delay but a phase advance in later phases of the firing cycle.

### Synaptic coupling

We investigate two models for pulse-coupling between the oscillators. For an instantaneous synapse with coupling strength ε*_ij_* the neuronal state *f_i_* of the MS phase oscillator after arrival of a spike from oscillator *j* is incremented instantaneously

(4) $$f_{i}^{\text{new}}\left(\varphi_{i}\right)=\min\left[f_{i}\left(\varphi_{i}\right)+\varepsilon_{ij},1\right],$$

with φ*_i_* the phase of the postsynaptic oscillator *i* at the time of the spike arrival. The phase φ*_i_* for which the oscillator reaches the threshold after spike input [i.e., *f_i_*(φ*_i_*) + ε*_ij_* = 1] is called the critical phase φ*_c_* (Zeitler et al., 2009) and is given by

(5) $$\varphi_{c}\left(\varepsilon_{ij}\right)=\frac{e^{b\left(1-\varepsilon_{ij}\right)}-1}{e^{b}-1}.$$

If a spike arrives at φ*_i_* < φ*_c_*, *f_i_* will increase instantaneously by ε*_ij_*. The instantaneous change of the state *f_i_* by ε*_ij_* corresponds to a phase shift $\Delta \varphi_{i}={f_{i}}^{-1}\left[f_{i}\left(\varphi_{i}\right)+\varepsilon_{ij}\right]-\varphi_{i},$ which yields

(6) $$\begin{array}{c} \Delta \varphi_{i}\left(\varphi_{i}\;\left|\;\varepsilon_{ij}\right.\right)=\left\{\;\begin{array}{ccc} \chi_{\text{b}}\left(\varepsilon_{ij}\right)+\beta_{b}\left(\varepsilon_{ij}\right)\varphi_{i} & \text{for} & 0\leq \varphi_{i}<\varphi_{c}\\ 1-\varphi_{i} & \text{for} & \varphi_{c}\leq \varphi_{i}<1 \end{array},\right. \end{array}$$

with

(7) $$\chi_{\text{b}}\left(x\right)\equiv \frac{\beta_{b}\left(x\right)}{\beta_{b}\left(1\right)}\text{, }\beta_{b}\left(x\right)\equiv e^{bx}-1,$$

where φ*_i_* is the phase of the postsynaptic oscillator just before arrival of the input spike. Note that φ*_c_*(ε*_ij_*) = [1 − χ*_b_*(ε*_ij_*)]/[1 + β*_b_*(ε*_ij_*)].

Tsubo et al. (2007) measured the phase shifts of layer-5 and layer-2/3 pyramidal neurons in rat motor cortex. The maxima of the averaged phase shifts were found to be in the second half of the oscillatory period for these neurons at all frequencies (including the gamma band; see their Figure 4). Hence we require here that the maximum of the phase shift Δφ*_i_* is in the second part of the intrinsic cycle of the oscillator, and therefore φ*_c_* > 0.5. This restriction imposes an upper bound of ε*_ij_* < 1 − ln[(1 + *e^b^*)/2]/*b* = 0.21 for *b* = 3 on the synaptic strength through Eq. 5. In case of ε*_ij_* = 0.21, a spike can cause an increase in the state variable *f* of about 21% of the difference between rest state *f* = 0 and the threshold value *f* = 1. For the HH neuron, the upper bound for the maximum synaptic conductance is 3.15 mS/cm^2^, which corresponds to an increase of 21% of (*V*_onset_ − *V*_rest_), since the resting potential *V*_rest_ and the onset of the action potential *V*_onset_ are near −65 and −50 mV, respectively.

A more realistic synaptic coupling model is provided by the so-called alpha function. For an “alpha synapse,” the postsynaptic potential after arrival of a spike at time *t*_0_ at the synapse with strength ε*_ij_* is described by

(8) $$\alpha \left(t\;\left|\;\varepsilon_{ij},\tau_{\text{syn}}\right.\right)=\left\{\;\begin{array}{ccc} 0 & \text{for} & t<t_{0}\\ \varepsilon_{ij}\frac{t-t_{0}}{\tau_{\text{syn}}^{2}}\exp\left(-\frac{t-t_{0}}{\tau_{\text{syn}}}\right) & \text{for} & t\geq t_{0} \end{array}\right.,$$

where τ_syn_ > 0 is the synaptic rise time of the input. Unless stated otherwise, τ_syn_ = 2 ms in this study. The numerical simulations are implemented using an Euler scheme with a time step size equal to 2.5 μs (≈10^−4^*T*_0_). In our implementation, there is no current reset after spiking of the postsynaptic neuron, i.e., the “tail” of the alpha function is carried over into the next cycle.

### Spike-timing dependent plasticity

In general, the synaptic coupling strength is not constant, but varies depending on pre- and post-synaptic activity due to STDP (Hebb, 1949; Bi and Poo, 1998). We have implemented the additive STDP rule (Froemke et al., 2006) for both instantaneous and alpha synapses. For a pre-synaptic spike at arrival time $t_{k}^{\text{arr}}$ and a postsynaptic spike at $t_{l}^{\text{spike}}$ the fractional synaptic modification *W*(Δ*t*) is given by

(9) $$W\left(\Delta t\right)\equiv \left\{\begin{array}{ccc} A_{-}\exp\left(\frac{\Delta t}{\tau_{-}}\right) & \text{for} & \Delta t<0\\ 0 & \text{for} & \Delta t=0,\\ A_{+}\exp\left(-\frac{\Delta t}{\tau_{+}}\right) & \text{for} & \Delta t>0 \end{array}\right.$$

with $\Delta t\equiv t_{l}^{\text{spike}}-t_{k}^{\text{arr}}.$

The spike arrival time $t_{k}^{\text{arr}}$ is defined as the time of the onset of the postsynaptic potential, as in the experimental protocol by Bi and Poo (1998). Unless stated otherwise, the time constants for potentiation τ_+_ and depression τ_−_ are τ_+,0_ ≡ 16.8 ms and τ_−,0_ ≡ 33.7 ms, respectively. In this study the standard values for the maximum amplitude of potentiation *A*_+_ and depression *A*_−_ are *A*_+,0_ ≡ 0.78 and *A*_−,0_ ≡ −0.27, respectively. These standard parameter values for STDP were fitted to the data from Bi and Poo (1998), who determined the fractional synaptic modification *W* as the relative change in synaptic strength after evoking an input and output spike pair 60 times. We assume that the change of the synaptic weight Δε caused by each input spike is constant and defined by Δε = ε_0_*W*(Δ*t*)/60 (Song et al., 2000; van Rossum et al., 2000) with initial synaptic weight ε_0_ and *W*(Δ*t*) as in Eq. 9. Therefore, the learning rule used here for a particular pair of pre- and post-synaptic spikes is given by ε_0_ → ε_0_ + ε_0_*W*(Δ*t*)/60, in agreement with Lee et al. (2009).

The simulation procedure of the network for STDP is as follows: The evolution of the state of the network is studied over 60 consecutive sessions. At the start of each session, the initial phases of the three oscillators are chosen arbitrarily from a uniform random distribution. The first session starts with equal coupling strengths for all synapses, which then change due to STDP. All succeeding sessions start with the coupling strengths that resulted at the end of the previous session, but with re-randomized phases of the oscillators to prevent that the system converges into a locally rather than a globally stable state.

In order to be physiologically relevant for synchronization, convergence should not take too much time. Therefore, we assume that convergence to a stable relative phase between oscillators 1 and 3 in the model takes place within a session consisting of *n*_sess_ = 15 cycles. Thus with STDP we typically run 60 sessions of 15 cycles each to investigate convergence of the network into a stable synchronization state. Without STDP the couplings do not change and a single session of 15 cycles is used. To avoid any spurious dependence on the random initial phases, we repeat each calculation 35^3^ = 45,875 times. Thus in the STDP case almost 40 million cycles are computed for every chosen setting of coupling strengths and delay times.

### Phase-locking equation and stability analysis

Phase-locking equations are useful to determine the new period of a network and the relative phases of coupled oscillators (van Vreeswijk et al., 1994; Bressloff and Coombes, 1998). For the simple MS phase oscillator, the phase-locking equation can be derived analytically. A full analysis for our model is provided in the Appendix, where we derive the relationships between synaptic weight, conduction delay, and the new period of the oscillators for zero-lag 1:1 phase-locked firing of the outer oscillators. Here we will just consider a simple example (see Figure 1B).

Without loss of generality, we set the time of the (*n*−1)-th firing of the relay neuron $t_{2,n-1}^{\text{spike}}=0$ and call the period of the 1:1 phase-locked oscillators *T*. For zero-lag synchrony there is a þeta ∈ [0, 1) such that the outer oscillators fire at $t_{1,n-1}^{\text{spike}}=t_{3,n-1}^{\text{spike}}=\theta T,t_{1,n}^{\text{spike}}=t_{3,n}^{\text{spike}}=\theta T+T,$ etc. For equal delays τ_1_ = τ_3_ = τ, both spikes from the outer oscillators arrive at the relay oscillator at the phase τ + θ*T*/*T*_0_ (if τ + θ*T*/*T*_0_ ≤ *T*/*T*_0_) with excitatory coupling strength ε. The two synaptic inputs reduce the period of the relay oscillator from the intrinsic period *T*_0_ to

(10) $$T=T_{0}\left[1-\Delta \varphi \left(\tau +\theta T/T_{0}|2\text{ε}\right)\right],$$

with Δφ as defined in Eq. 6. Since the relay neuron fires at *T*, the outer oscillators will each receive a spike at *T* + τ*T*_0_. The outer neurons spiked at θ*T* and hence their phase will be τ + (1 − θ)*T*/*T*_0_ (if τ + (1 − θ)*T*/*T*_0_ ≤ *T*/*T*_0_) at the arrival of the spike from the relay neuron. Therefore the period of the outer oscillators is given by

(11) $$T=T_{0}\left[1-\Delta \varphi \left(\tau +\left(1-\theta \right)T/T_{0}|\text{ε}\right)\right].$$

Equations 10 and 11 are the 1:1 phase-locking equations for the mentioned conditions.

Assume now that both arrival phases exceed the critical phases φ*_c_*(2ε) and φ*_c_*(ε), respectively. Then rewriting Eqs 10 and 11 using Eq. 6 yields

(12) $$T=\tau T_{0}+\theta T\text{ and }T=\tau T_{0}+\left(1-\theta \right)T\Rightarrow T=2\tau T_{0},\theta =1/2.$$

**Figure 2:**
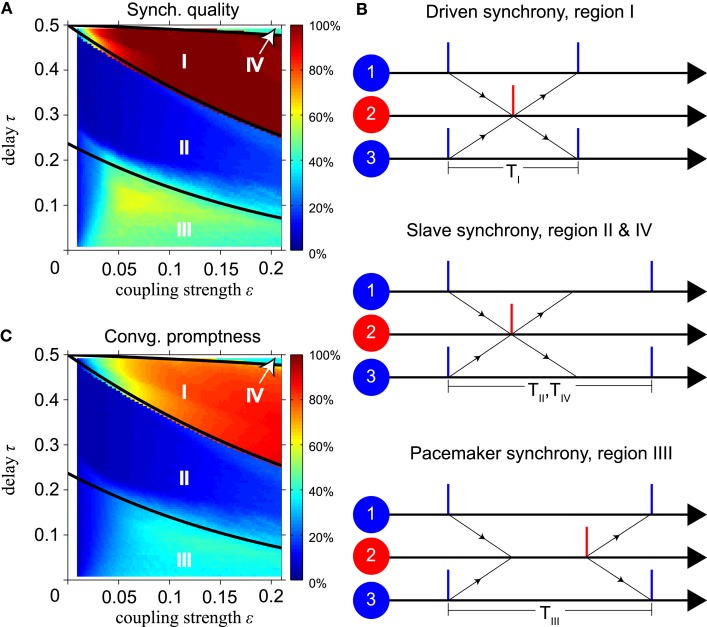
**Features of synchronization depending on delay time and coupling strength**. **(A)** Synchronization quality (SQ) and **(C)** convergence promptness (CP) for model with instantaneous synapses. Thick black lines in **(A,C)** indicate boundaries of dynamics calculated analytically using the phase-locking equation. **(B)** Illustration of synchrony modes dominant in regions indicated by roman numerals in **(A,C)**. SQ is high in region I, low in regions II and IV, and intermediate in region III. Slave synchrony (regions II and IV) is not stable, whereas driven (region I) and pacemaker (region III) synchrony are asymptotically stable. CP is highest in region I, indicating synchronization within about four cycles.

Thus the period of all oscillators is twice the conduction delay and activity switches between inner and outer oscillators every half period. All oscillators immediately spike upon spike input. We will call this mode the “driven synchrony” (DS) mode (see Figure 2B). Two other typical modes are also illustrated in Figure 2: “slave synchrony,” where only the relay oscillator spikes directly after input, and “pacemaker synchrony,” where only the outer oscillators do so.

To investigate the stability of DS we assume small perturbations. Since we use the relay neuron spikes as reference time, the perturbation affects the phase of the outer oscillators to (τ + δφ_1_, τ + δφ_3_) at $t_{2,n-1}^{\text{spike}}.$ DS is asymptotically stable, if there is a δ > 0 such that the phases of the outer oscillators 1 and 3 will be closer to τ at the next spike at $t_{2,n}^{\text{spike}}$ for $\sqrt{\delta \varphi_{1}^{2}+\delta \varphi_{3}^{2}}<\delta$. Since φ*_c_*(ε) < 2τ, we can define a value δ ≡ 2τ − φ*_c_*(ε) > 0, and thus φ*_c_*(ε) = 2τ − δ. The spike from the relay arrives when the phase of oscillator 1 is in the range 2τ − δ < φ_1_ < 2τ + δ, which exceeds this critical phase φ*_c_*(ε). Therefore oscillator 1 will spike immediately after receiving input from the relay neuron, and the same is true for oscillator 3. Thus perfect synchronization is re-established as long as the perturbation was sufficiently small, proving that the DS mode is an asymptotically stable mode. To determine the stability of other synchrony modes it is necessary to calculate the eigenvalues λ of the Jacobian of the (phase) return map (Zeitler et al., 2009), see the Appendix for details.

We now briefly consider the effect of STDP on slave synchrony. The coupling strengths from the outer oscillators to the relay oscillator remain unchanged for slave synchrony, since the relay oscillator immediately spikes upon input from the outer neurons. In the Appendix we show that the coupling strengths from the relay oscillator to the outer oscillators increase with ε*_n_* > ε*_n - 1_* for τ ≥ 0.25 using the STDP window *W*(Δ*t*) of Eq. 9, since the spike from the relay oscillator arrives well before the outer neurons spike. Hence synaptic weights increase to $\varphi_{c}^{-1}\left(2\tau \right)=1-\text{ln}\left[2\tau \left(e^{b}-1\right)+1\right]/b$, at which point the outer oscillators also spike immediately after input and STDP stops. Thus STDP turns slave synchrony into DS.

**Figure 3:**
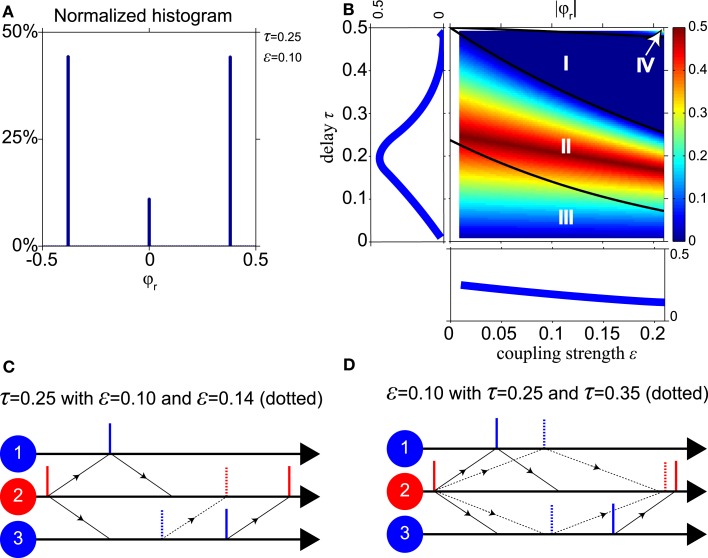
**Dominance of non-zero relative phases**. **(A)** Normalized histogram of the relative phase φ*_r_* for stable states with τ = 0.25 and ε = 0.1. For about 90% of possible initial phases, the system converges to a non-zero relative phase. **(B)** |φ*_r_*| As function of the conduction delay τ and the synaptic weight ε, with averages projected into side panels. |φ*_r_*| decreases when ε increases, and has a maximum for τ near 0.2. **(C,D)** Dynamics of “pacemaker-slave” synchrony corresponding to non-zero relative phase. Solid lines represent spike times for parameter values τ = 0.25 and ε = 0.1, dotted lines for a stronger synaptic weight ε = 0.14 in **(C)** and longer delay time τ = 0.35 in **(D)**, respectively.

In the driven, pacemaker, and slave synchrony modes oscillators 1 and 3 spike simultaneously. There are other, asynchronous stable modes where this is not the case. Figures 3C,D show the dynamics of the oscillators for a typical asynchronous case. This mode will be referred to as a “pacemaker-slave” because the relay drives oscillator 1 and is driven by oscillator 3.

### Synchronization measures

For a proper analysis of synchronized firing, a quantitative definition of synchrony is required. In experiments perfect synchrony will never be observed as noise causes small variations in the timing of action potentials. Instead we will define synchrony as firing of two oscillators within a small time window, where the time window should be sufficiently large to eliminate the effect of noise, and sufficiently small to provide an accurate estimate of synchrony. While we could measure synchrony here to the limits of the numerical accuracy of our simulations, we define synchrony as the condition when the spikes of the outer oscillators occur within $\left|\;t_{1,n}^{\text{spike}}-t_{3,n}^{\text{spike}}\right|\leq 0.02T_{\text{0}}$ (Engel and Singer, 2001), which is 0.5 ms for simulations of the model with MS phase oscillators and 0.2932 ms for the model with the classical HH neurons.

Synchronized firing of the outer oscillators does not only depend on the synaptic weights and delay times, but also on the initial phase of the three oscillators. Hence we have to consider the robustness of convergence to synchronous firing for variations in the initial relative phase of the neuronal oscillators. Therefore, we define “*synchronization quality*” (SQ) as the fraction of the number of initial phase combinations that leads to stable synchronous firing of the outer oscillators. SQ has a value between 0 and 100%, where 100% means that the outer oscillators will always converge to synchronous spiking within the simulation period, independent of their initial phases. This provides a measure of the attraction domain of the initial phases for reaching synchronization of the outer oscillators. To determine SQ, we repeat our simulations 35^3^ = 42,875 times with randomly chosen initial phases.

In order to investigate the impact of the various parameters of the STDP learning window, we wish to derive a single value for the SQ, rather than one value for each combination of synaptic weights and conduction delay. For this purpose we consider various synaptic weights in 100 evenly spaced steps in the range from 0.01 to 0.21 and conduction delays in 100 evenly spaced steps in the range from 0.01 to 0.49. Then we average the SQ over these 10,000 pairs to obtain an “average SQ” for each parameter set of the learning window. Note that for an STDP run with 60 sessions, this means that we compute almost 400 billion cycles of the model for each change of the STDP parameters.

For some combinations of the synaptic weights, delay times, and initial phases the state of zero-lag synchronization is reached faster than for other combinations. Therefore, we define a “*convergence promptness*” (CP) for the network to achieve zero-lag synchronization of the outer oscillators. This is calculated as CP = SQ · (1 − 〈*n*_sync_〉/*n*_sess_), where 〈*n*_sync_〉 is the average number of intrinsic periods *T*_0_ needed to achieve zero-lag synchrony. When there is no zero-lag synchrony established within *n*_sess_ = 15 cycles, CP equals to 0. Note that 〈*n*_sync_〉 ≤ *n*_sess_ with *n*_sess_ = 15 for the simulations in this study, and that the measure is multiplied with the SQ to account for the readiness of the system to achieve zero-lag synchrony at all.

Finally, not only are we interested in synchrony of the outer oscillators, but also in the relative phase φ*_r_* for stable modes in which the oscillators 1 and 3 are not in synchrony. The relative phase between the outer oscillators is defined by φ*_r_* ≡ $\left(t_{3,n}^{\text{spike}}-t_{1,n}^{\text{spike}}\right)/T_{0}$ with a value between −1 and 1. Since values for φ*_r_* of −1, 0, and 1 all refer to the same state, we report φ*_r_* rescaled to the range −1/2 to 1/2 by periodically mapping [−1, −0.5] to [0, 0.5] and [0.5, 1] to [−0.5, 0], respectively.

## Results

### Equal delay times and coupling strengths

Figures 2A,C show simulation results for the SQ and the CP, respectively, as a function of the conduction delay τ and synaptic weight ε for the MS model with instantaneous synapses, identical delay times (τ_1_ = τ_3_ = τ) and identical coupling strengths (ε_12_ = ε_21_ = ε_23_ = ε_32_ = ε). The three solid black lines show the analytically calculated boundaries between different modes of synchrony using the phase-locking equations, for details see Section “Synchrony Modes Derived from the Phase-Locking Equation” in the Appendix. The upper right area, indicated by IV, is bounded by the line

(13) $$\tau =\frac{1-\chi_{b}\left(\varepsilon \right)}{2},$$

where χ*_b_* is defined in Eq. 7. For a detailed derivation see the discussion below Eq. A12 in Appendix. Our simulations show that in region IV zero-lag synchronization occurs mainly when the relay oscillator spikes immediately upon arrival of synaptic input from the outer neurons, but the outer neurons do not after input from the relay neuron, see the middle panel of Figure 2B. The period *T*_IV_ of this “slave synchrony” mode is given by Eq. A12 “SS2” in Appendix.

The line separating regions I and II is given by τ = φ*_c_*(ε)/2. In region I, zero-lag synchronization occurs mainly when both the relay and outer oscillators spike immediately when synaptic input arrives, see the upper panel in Figure 2B. This represents “DS” with period *T*_I_ for all neurons, see Eq. A7 in Appendix. The line separating regions II and III is given by

(14) $$\tau =\frac{1-\chi_{b}\left(2\varepsilon \right)-\varphi_{c}\left(\varepsilon \right)}{2\beta_{b}\left(2\varepsilon \right)}$$

where φ*_c_*, χ*_b_*, and β*_b_* defined in Eqs 5 and 7, respectively. For a detailed derivation see the discussion below Eq. A10 in Appendix. Region II shows mainly slave synchrony like region IV, but with a different period *T*_II_, see Eq. A12 “SS1” in Appendix. In region III, zero-lag synchronization occurs mainly when the outer oscillators spike immediately upon arrival of synaptic input while the relay oscillator does not. This “pacemaker synchrony,” cf. the lower panel in Figure 2B, has period *T*_III_ according to Eq. A10 “PS1” in Appendix.

Figure 2A shows that the SQ changes from high (region I) to low (region II) and back to intermediate values (region III) when the conduction delay decreases from 0.5 to 0. Stability analysis indicates that regions II and IV show poor SQ because slave synchrony, which dominates in these regions, is unstable, whereas driven and pacemaker synchrony, which dominate in region I and III, are asymptotically stable and robust against changes in the initial phases. Most combinations of delay and synaptic weight yield a SQ that is below 50%. Figure 2C shows that the delay and the synaptic weight in region I yield fast convergence to synchronization within about four cycles. In the other regions, zero-lag synchrony is established much more slowly or not at all.

Figure 3A shows a histogram of the relative phase φ*_r_* for the stable pacemaker-slave (φ*_r_* ≠ 0) and unstable slave synchrony (φ*_r_* = 0) modes of the model with a conduction delay τ = 0.25 and a synaptic weight ε = 0.1 (parameters in region II, cf. Figure 2A). Two non-zero relative phases, corresponding to the stable pacemaker-slave mode, and one zero-lag phase, corresponding to the unstable slave synchrony mode, occur. The non-zero relative phases have the same absolute value because of the symmetry of the network. For about 90% of the initial phases of the oscillators the system converges to the two non-zero relative phases and for 10% to a state with zero-lag synchrony. For other values of the delay and the weight in region II, III, and IV, the histograms are qualitatively similar to that shown in Figure 3A, i.e., two non-zero and one zero-lag relative phase. Thus in general zero-lag synchrony only occurs for a very limited set of initial phases in parameter regions II, III, and IV.

To illustrate how the non-zero relative phase varies for different parameter values in the network, Figure 3B shows the absolute value |φ*_r_*| as a function of the conduction delay τ and the synaptic weight ε. The three black lines in Figure 3B indicate the boundaries between the regions I, II, III, and IV as in Figure 2 calculated analytically using the phase-locking equation. To understand the results in Figure 3B, we consider the “pacemaker-slave” mode that corresponds to the positive relative phase illustrated in Figures 3C,D. Vertical solid lines represent the spiking times for ε = 0.1 and τ = 0.25, and the dotted lines correspond to a stronger weight ε = 0.14 in Figure 3C and a longer delay τ = 0.35 in Figure 3D, respectively. When the synaptic weight increases in Figure 3C, oscillators 2 and 3 will spike sooner after input from oscillator 1, whereas oscillator 1 always spikes immediately after input from oscillator 2. Hence the relative phase between oscillators 3 and 1 decreases when the coupling strength increases. At longer delay times in Figure 3D, the synaptic inputs from oscillator 2 arrive later in time at oscillators 1 and 3 as indicated by the dotted arrows. The input causes oscillator 1 to spike immediately, but not so for oscillator 3. For the longer delay time, oscillator 3 will be further in its natural cycle and therefore it will spike sooner after the input. Thus oscillators 1 and 3 will both spike later, but their relative phase difference is reduced for longer delays. The left panel of Figure 3B shows the relative phase as a function of the delay, averaged over all values of synaptic coupling. The lower panel shows the relative phase as a function of coupling strength, averaged over all delays.

**Figure 4:**
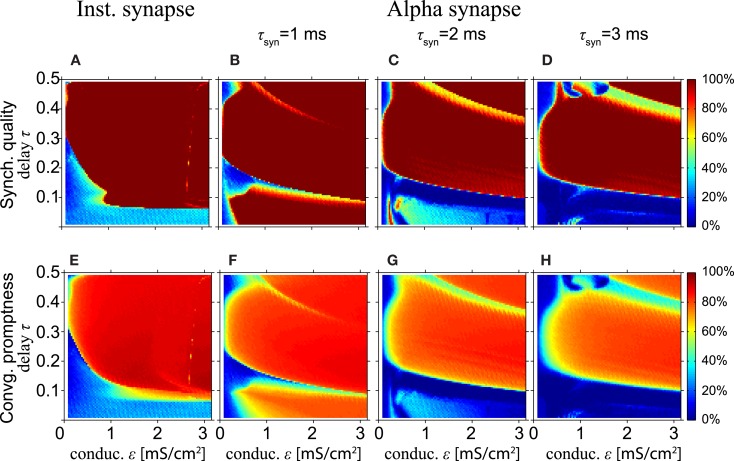
**Excellent synchronization for alpha synapses with short rise times**. (Top row) Synchronization quality (SQ) and (bottom row) convergence promptness (CP) for a Hodgkin–Huxley based model with instantaneous synapses **(A,E)** and alpha synapses for various rise times **(B–D,F–H)**. Comparing **(A,E)** with the results for the MS neurons (see Figures 2A,C) shows that SQ and CP clearly improve for HH neurons. Moreover, SQ and CP for short rise times of the alpha synapses **(B,F)** are better than for instantaneous synapses **(A,E)**, but decrease for longer rise times.

Next, we study synchronization for a model with HH neurons. Figures 4A,E show simulation results for the model with HH neurons for the SQ and the CP, respectively, as a function of the conduction delay τ and synaptic weight ε for instantaneous synapses. Comparing Figures 4A,E with the same results for the MS oscillator in Figures 2A,C shows that the model with HH neurons yields a high SQ and large CP for a larger range of τ and ε values than the MS neuron.

Figure 4A also shows that perfect SQ is not possible when the delay time is shorter than approximately 0.05 of the intrinsic period, which roughly corresponds to half the refractory period of the HH neuron. If the time delay is 0.05*T*_0_ or less, the time interval from spiking of the outer neurons and spike input from the relay neurons to the outer neurons is less or equal to the refractory period. In that case, input from the relay neuron to the outer neurons arrives in the refractory period, which effectively reduces the coupling strength when the outer neurons tend to synchronize and thereby disables the zero-lag DS mode when the delay time is short.

Figures 4B–D,F–H show the SQ and CP, respectively, as a function of the conduction delay τ and the synaptic weight ε for the HH neuron model with alpha synapses of various synaptic rise times (left, middle, and right columns for τ_syn_ = 1, 2, and 3 ms, respectively). Quite surprisingly, in the context of the results in Figures 4A,E, the SQ is high for very small time delays for τ_syn_ = 1 ms (Figure 4B). This can be understood from the fact that the alpha synapse adds an effective delay such that spike input from the relay neuron to the outer neurons arrives after the refractory period when the outer neurons fire in perfect synchrony. When the synaptic rise time increases, the range of time delays and synaptic strengths with high SQ decreases, cf. Figures 4B–D. To understand this, assume that synaptic inputs from the relay oscillator arrive when the phase of oscillators 1 and 3 is φ_1_ and φ_1_ + Δ, respectively. If Δ differs from zero, then oscillators 1 and 3 will spike at different times, unless the input is strong enough to immediately initiate a spike in both oscillators. For larger rise times and the same synaptic strength ε, less input per unit of time is received, since the synaptic input is spread out over a longer time. Therefore, input from an instantaneous synapse (equivalent to “τ_syn_ = 0”) or fast alpha synapse (small τ_syn_) can more readily synchronize the outer oscillators than a slow alpha synapse (large τ_syn_).

**Figure 5:**
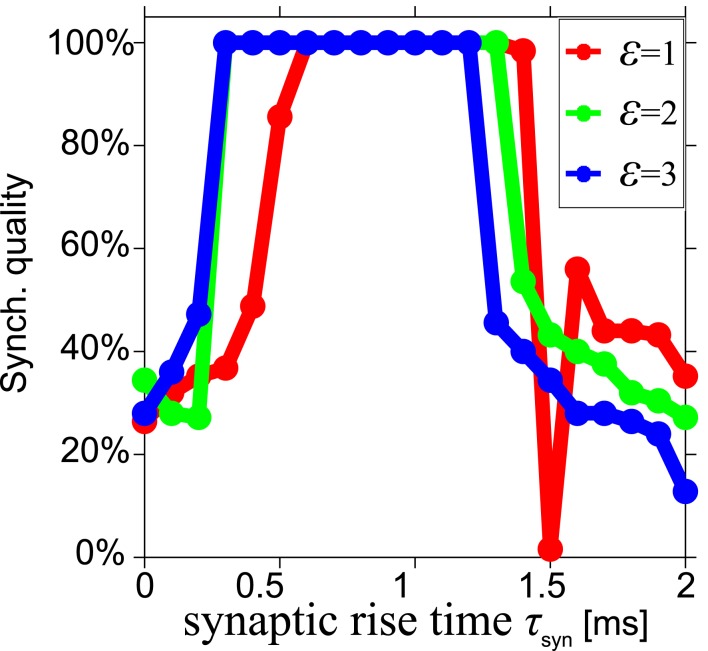
**Optimal synchronization for moderately fast synaptic rise time**. Synchronization quality at τ = 0.02 for three synaptic strengths ε = 1 (red), 2 (green), and 3 (blue) mS/cm^2^ in a model with alpha synapses and Hodgkin–Huxley neurons, shown as a function of the synaptic rise time τ_syn_. Almost perfect synchronization quality can be obtained when the synaptic rise time is moderately fast, i.e., approximately between 0.5 and 1 ms.

High SQ is possible for short delay times (τ < 0.05), if the synaptic rise time is short (Figure 4B), but less so when the synaptic rise time increases (Figures 4C,D). This suggests that there is a range of synaptic rise times which favor a high SQ for short time delays. Figure 5 shows the SQ, as a function of the synaptic rise time τ_syn_, evaluated at τ = 0.02 for ε = 1 (red), 2 (green), and 3 (blue) mS/cm^2^. Perfect SQ is obtained when the synaptic rise time is moderately fast, i.e., approximately between 0.5 and 1 ms, for a large range of synaptic coupling strengths. Therefore, moderately fast synaptic rise times favor zero-lag synchrony.

### STDP facilitates zero-lag synchronization

**Figure 6:**
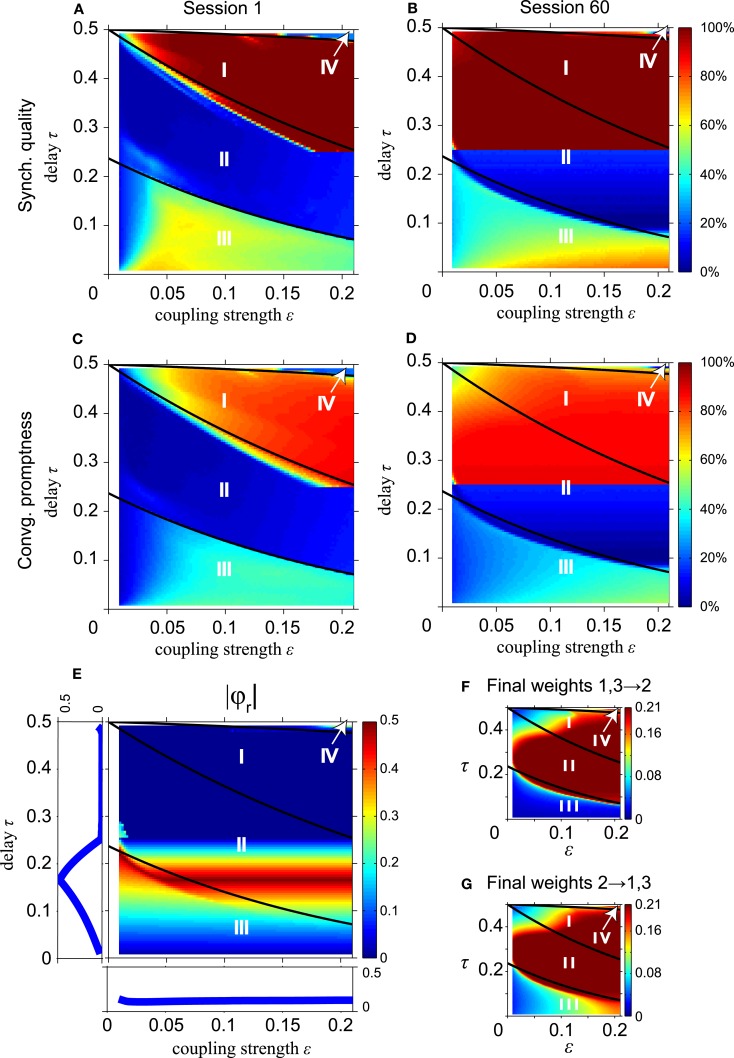
**STDP improves long-range synchronization for instantaneous synapses and decreases the fraction of non-zero relative phases**. **(A,B)** Synchronization quality after the first and the 60-th session, respectively. **(C,D)** Corresponding convergence promptness. For conduction delays τ ≥ 0.25, synchrony improves due to an increase of initial weights ε by STDP. **(E)** |φ*_r_*| as in Figure 3B but after 60 learning sessions. **(F)** Synaptic weights from the outer oscillators to the relay oscillator; **(G)** synaptic weights from the relay oscillator to the outer oscillators. Changes in |φ*_r_*| relative to that in Figure 3B are largely caused by an increase of the synaptic weights by STDP. To compare with the results without STDP, we show the solid black lines which separate regions with different dynamics in Figure 2.

We now investigate the effect of STDP on synchronization of the MS neuron, starting with the simple instantaneous synapses. Figure 6 shows the SQ and CP with short (left column) and long STDP adaptation (right column), i.e., after the first and sixtieth session, respectively. To allow an easy comparison to the results without STDP in Figure 2, we have drawn the same thick black lines in Figure 6 which separate regions with different dynamics as in Figure 2. Note that the synaptic strength ε along the horizontal axes in Figure 6 represents the *initial* synaptic weight, not the final values after adaptation by STDP. The results in Figures 6A,B show that the effect of STDP is a gradual expansion of the range of coupling strengths which give rise to synchronization. With STDP, the weak coupling gradually increases to larger synaptic strengths that allow synchronous firing, corresponding to DS. Likewise, the CP increases, see Figures 6C,D.

The higher SQ and the faster CP in region II for large delay times in Figures 6A–D can be understood as follows: by increasing coupling strengths STDP converts slave synchrony, which is unstable and a dominant zero-lag synchronization mode in region II, into DS, which is asymptotically stable. For slave synchrony STDP increases the coupling strength from the relay oscillator to the outer oscillator. The sharp border is related to the maximum coupling strength ε_max_ = 0.21: the slave to drive synchrony conversion can only occur if τ exceeds $\tau_{c}^{+}\equiv \varphi_{c}\left({\text{ε}}_{\text{max}}\right)/2\approx 0.25.$ Notice that the fully improved domain of attraction for zero-lag synchrony is reached only after 60 sessions with STDP. This suggests that STDP can contribute to zero-lag synchrony, but generally only after many cycles of weight adaptation (here up to 900 oscillations).

**Figure 7:**
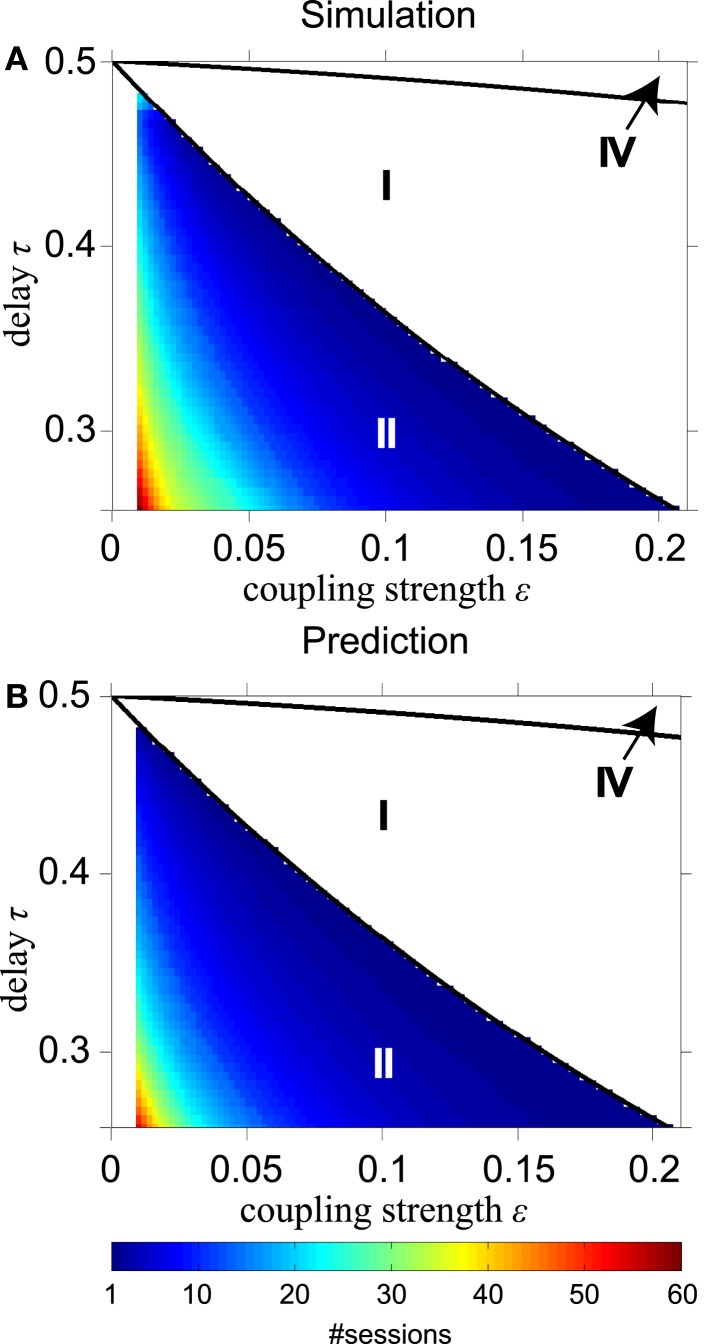
**Time required to reach 100% synchronization quality by STDP**. **(A)** Number of sessions to achieve 100% synchronization quality according to direct simulations; **(B)** same according to analytical calculations for converting slave into driven synchrony for the relevant part $\varphi_{c}^{+}\leq \tau <\varphi_{c}\left(\varepsilon \right)∕2$ of region II. Solid black lines in **(A,B)** separate regions with different dynamics as shown in Figure 2.

Figure 7 shows the number of sessions for STDP required to obtain 100% SQ, if the network begins with four equally strong coupling strengths in the range between 0 < ε ≤ 0.21 and delay times $\tau_{c}^{+}\leq \tau <\varphi_{c}\left(\text{ε}\right)/2,$ i.e., where STDP changes slave synchrony into DS as just discussed. The solid black lines in Figure 7 separate the regions with different dynamics as in Figure 2 (without STDP). Figures 7A,B are obtained from direct simulations and from analytical calculations, respectively. The analytical results are obtained using Eqs A26 and A27 in Appendix iteratively. The simulated and analytical results are in good agreement. The network requires a slightly smaller number of sessions to reach a high SQ value in the analytical calculations, because these start from the condition of slave synchrony, whereas the direct simulations start from random initial phases and reach the driven state after achieving slave synchrony first. For smaller initial coupling strengths it takes more time to converge to stable zero-lag synchronization with STDP: 20 cycles (about 0.5 s) or more.

Figure 6E shows the absolute value of the non-zero relative phase |φ*_r_*| as a function of the delay time τ and the synaptic weight ε after convergence of the network to a stable state by STDP. This result should be compared to the case without STDP in Figure 3B. Note that the synaptic strength ε along the horizontal axes in Figure 6 represents the initial synaptic weight at the start of the simulations, which is assumed to be equally strong for the four connections (cf. Figure 1A) and will change during the simulations by STDP. The average values of final synaptic weights are shown as a function of the delay and the weight in Figure 6F (connections from the outer to the relay oscillator) and Figure 6G (connections from the relay to the outer oscillators), respectively. After training for 60 sessions, the values of |φ*_r_*| for the stable states of the network, shown in Figure 6E, significantly decrease in region II while |φ*_r_*| changes slightly in regions I, III, and IV as compared to the values before adaptation of the synapses by STDP (Figure 3B). This is largely caused by STDP-induced increase of the coupling strength in region II, as shown in Figures 6F,G. The final weights overall are quite similar in both directions in region II. In region III they differ because pacemaker synchrony is dominant, which implies different timing of pre- and post-synaptic firing for the relay and outer neurons, and therefore different effects of STDP. The final weights in regions I, II, and IV in Figures 6F,G are similar because, after many learning sessions, DS is dominant here. In DS, the firing behavior of the relay and outer oscillator is the same, i.e., a synaptic input arrives at phase 2τ and makes the neuron spike instantaneously. Since all connections start with the same initial weight, the same weight adaptation is applied to the connections from the relay to the outer oscillators and vice versa.

**Figure 8:**
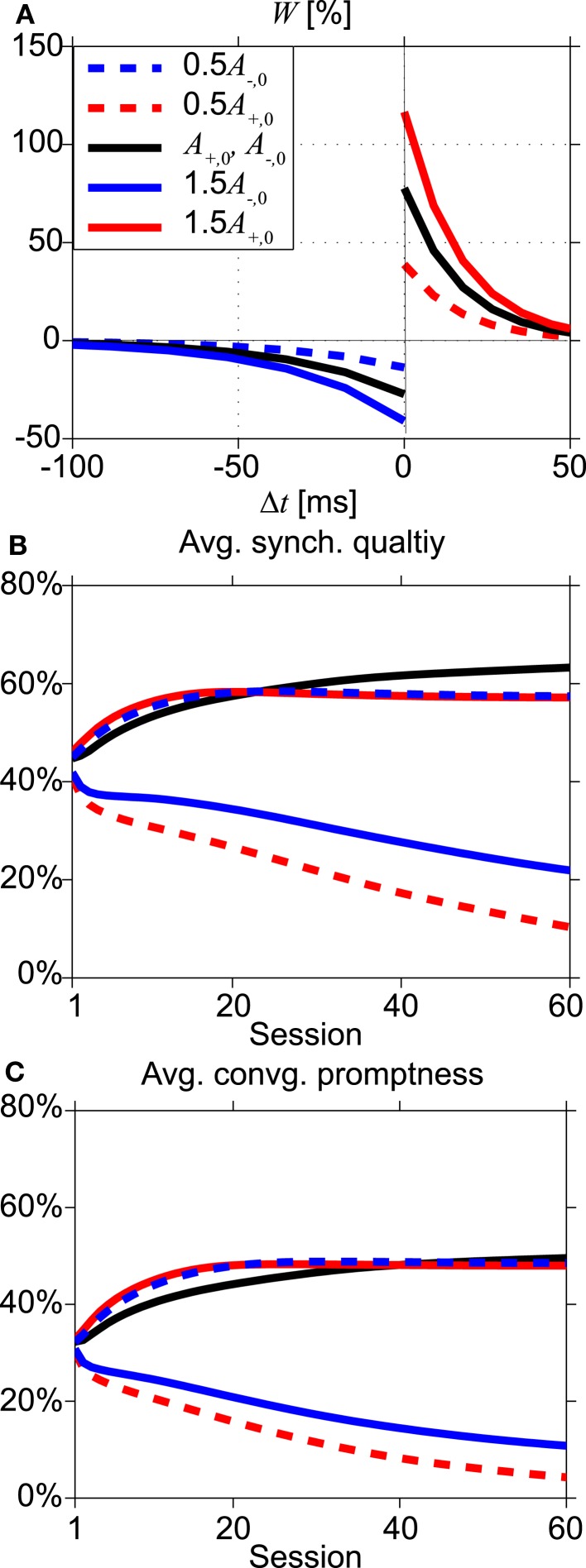
**Effects of changing the STDP learning window**. **(A)** Shapes of the learning windows for various amplitudes of *A*_+_ and *A*_−_. Black lines represent the standard parameter set (Bi and Poo, 1998), while red and blue lines indicate changing values of potentiation *A*_+_ and depression *A*_−_, respectively, by a factor 0.5 (dashed lines) or 1.5 (thick lines). **(B,C)** Dependence of average synchronization quality and convergence promptness, respectively, on the number of learning sessions. After about 25–40 sessions, the standard learning window (black lines) becomes optimal.

In order to investigate the robustness of our results for variations of the STDP parameter values, we return to the instantaneous synapses. We have varied the amplitudes *A*_+_ and *A*_−_ of the fractional synaptic modification *W*, see Eq. 9 and Figure 8A. Figures 8B,C show the SQ and the CP, respectively, for various values of *A*_+_ and *A*_−_ as a function of the number of learning sessions. The black lines in Figure 8 show the results for the asymmetric learning window with our standard parameters of STDP obtained from the Bi and Poo (1998) data, while the red lines show results for smaller (0.5*A*_+,0_: dashed line) and larger (1.5*A*_+,0_: thick line) values of potentiation amplitude *A*_+_. The blue lines are for smaller (0.5*A*_−,0_: dashed line) and larger (1.5*A*_−,0_: thick line) values of depression amplitude *A*_−_.

When depression dominates over potentiation (dashed red and solid blue lines), the SQ is poor even after many sessions, see Figure 8B. Larger values for potentiation relative to depression give rise to faster and better synchrony (dashed blue and solid red lines). However, after about 25–40 learning sessions, the standard set of parameters (Bi and Poo, 1998) yields better results, i.e., higher values of average SQ and faster convergence to synchronization, see Figures 8B,C. To explain this, we will consider why the average SQ in region III becomes higher for the standard set of STDP parameters than for the potentiation-dominated parameter sets (dashed blue and solid red lines in Figure 8A). For the other regions (I, II, and IV) both parameter sets yield similar values of average SQ, because there the system converges to the DS mode after several learning sessions.

We first consider the effects of STDP on the coupling strengths of the network starting with initial coupling strength ε and delay time τ in region III just below the line which separates region II and III. STDP will increase the weights ε to larger values due to larger potentiation relative to depression. This moves (ε, τ) from region III to region II, where the zero-lag synchronization modes “SS1” of Eq. A12 in Appendix and “NDS2” of Eq. A13 in Appendix are unstable. Hence the average SQ in region III decreases because the initial weights ε will be increased to values that correspond to unstable zero-lag synchronization modes in region II. However, when the initial weights and the delay times are farther away from the line separating region II and III, the effects of STDP on the weights are different. To understand this, we focus on pacemaker synchrony, a stable synchronization mode in region III. The period of oscillations for pacemaker synchrony in region III becomes longer when the weights ε become smaller, since the delay between the arrival of spike input and firing increases, cf. formula “PS1” in Eq. A10 in Appendix. Since the time window for depression is much larger than that for potentiation, longer delays suppress potentiation more, leaving depression dominant. Close to pacemaker synchrony, this concerns mainly the synapses from the outer oscillators onto the relay neuron, since the outer neurons fire quickly after receiving input. A weakening of these synapses reinforces pacemaker synchrony. Overall then, increasing the strength of potentiation beyond the standard rule will shift more (ε, τ) to region II without stable synchronization, leaving fewer (ε, τ) that will achieve stable pacemaker synchrony. Hence the SQ and CP in region III eventually becomes higher for the standard STDP set than for the potentiation-dominated one; and the average over all regions follows this trend, see Figures 8B,C.

**Figure 9:**
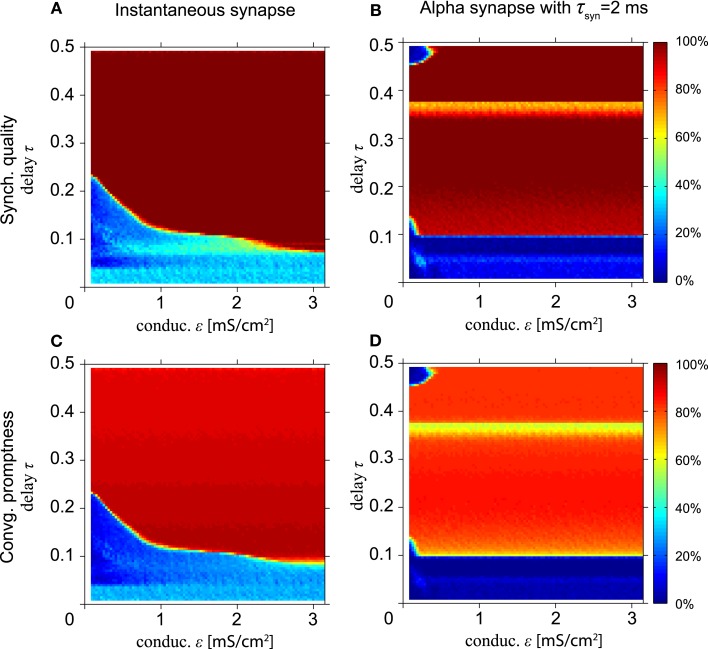
**Spike-timing dependent plasticity improves long-range synchronization for the model with HH neurons**. **(A,B)** Synchronization quality after the 60-th session for instantaneous synapses and the τ_syn_ = 2 ms alpha synapse, respectively. **(C,D)** Corresponding convergence promptness. For conduction delays approximately τ ≥ 0.1, synchrony improves due to STDP-mediated increase of synaptic strength ε.

Results for different time constants for potentiation τ_+_ and depression τ_−_ (not shown) are qualitatively similar to the results for changing the amplitudes *A*_+_ and *A*_−_, i.e., a larger value of τ_+_ relative to τ_−_ gives rise to faster and better synchrony, and the standard values for τ_+_ and τ_−_ yield better results for zero-lag synchrony when the number of the learning sessions is large. Our results are robust for decreases of depression (dashed blue line) or increases in potentiation (solid red line), but not vice versa (dashed red and solid blue lines). Figure 9 shows the SQ and CP for the model with STDP with HH neurons with instantaneous synapses (Figures 9A,C, respectively) and with alpha synapses (Figures 9B,D). Comparing Figure 9A with Figure 4A reveals hardly any improvement of the SQ by STDP. However, for alpha synapses STDP significantly improves the average SQ by increasing the synaptic coupling ε (compare Figures 9B and 4C).

In summary, the conditions that contribute to zero-lag long range synchronization are as follows. First, the delay times relative to the intrinsic frequency *T*_0_ should be long enough, i.e., more than about 0.35 (8.75 ms for *T*_0_ = 25ms) for the model with MS phase oscillators with the instantaneous synapse (cf. Figures 2A,C) and 0.15 (2.2 ms for *T*_0_ = 14.66 ms) for the model with HH neurons with the alpha synapse (cf. Figure 4). Moreover, alpha synapses with short synaptic rise times are required for zero-lag synchronization for short delay times (cf. Figure 5). Third, STDP facilitates the synchronization but generally only after many cycles of weight adaptation (cf. Figures 6–9).

### When delay times from the relay neuron to outer neurons are not identical

We now investigate the dynamics of the model in Figure 1A, when the delay times τ_1_ and τ_3_ are different. Simulation results (not shown) show that zero-lag synchrony then is lost, in agreement with Fischer et al. (2006) and Vicente et al. (2008). Similar results (not shown) were found for the model with HH neurons. To understand why zero-lag synchronization disappears when the delay times are different, we consider the MS network with instantaneous synapses. Let us assume that oscillators 1 and 3 fire simultaneously. When oscillator 2 generates an action potential, the spikes arrive at oscillators 1 and 3 after delays τ_1_ and τ_3_, respectively. When the spikes arrive at oscillators 1 and 3 at a phase exceeding the critical phase, oscillators 1 and 3 will spike immediately after arrival of the spike. This implies that oscillators 1 and 3 spike with a time difference $\left|\;t_{1,n+1}^{\text{spike}}-t_{3,n+1}^{\text{spike}}\right|=\left|\tau_{\text{1}}-\tau_{3}\right|\;T_{\text{0}}$. This illustrates that zero-lag synchrony is lost when the delay times τ_1_ and τ_3_ are different. Qualitatively similar results (not shown) are obtained for asymmetric coupling strengths.

**Figure 10:**
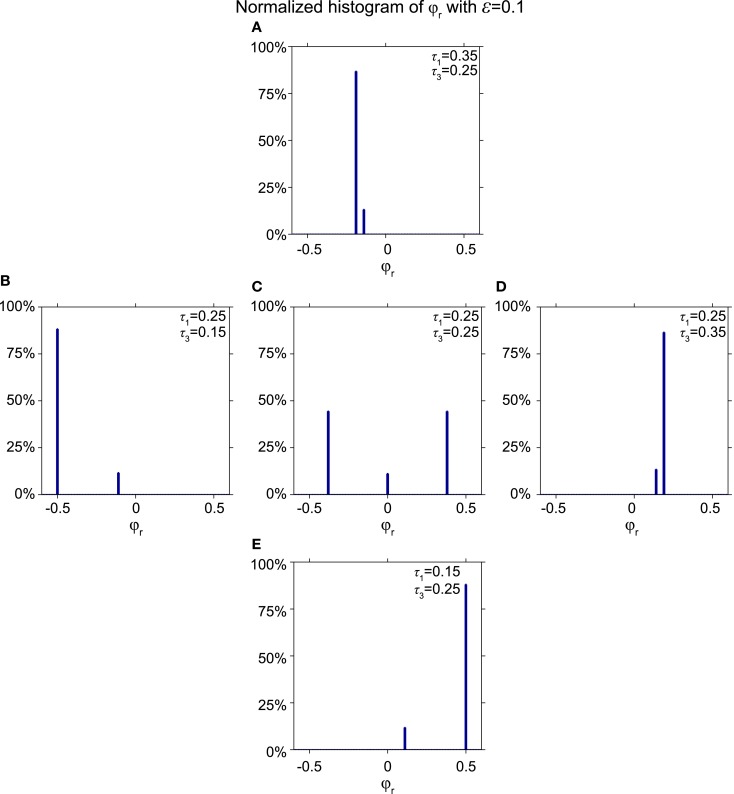
**Effect of unequal delays on relative phase**. **(A–E)** Histograms showing the fraction of initial phases that converge to a particular relative phase ε = 0.1 for different pairs (τ_1_, τ_3_) = (0.35, 0.25), (0.25, 0.15), (0.25, 0.25), (0.25, 0.35), and (0.15, 0.25). When delays between the outer oscillator and the relay neuron become asymmetric, one of the non-zero relative phases disappears and the zero relative phase shifts to non-zero values, corresponding to a state where the outer oscillator with the shorter delay spikes earlier.

Figure 10 shows a histogram of the relative phase φ*_r_* for various stable modes for the synaptic weight ε = 0.1 for five pairs of conduction delays: (τ_1_, τ_3_) = (0.35, 0.25) in Figure 10A, (0.25, 0.15) in Figure 10B, (0.25, 0.25) in Figure 10C, (0.25, 0.35) in Figure 10D, and (0.15, 0.25) in Figure 10E. Figure 10A shows two peaks of relative phases near −0.19 and −0.14, instead of the three peaks appearing in Figure 10C, which include one at zero. Figures 10B,D,E also yield two non-zero peaks instead of three. Figures 10A,B are mirror images of Figures 10D,E, respectively, because the delay times are exchanged and the outer neuron with the shorter delay time spikes first. Essentially, the symmetric non-zero peaks in Figure 10C collapse into one for asymmetric delays, and the zero-lag peak shifts to a non-zero value, which is equal to the value of the zero-lag peak. Other combinations of the synaptic weight ε and non-equal conduction delays τ_1_ and τ_3_ yield qualitatively similar results.

## Discussion

In this study we have investigated the conditions for zero-lag synchrony between two neuronal oscillators, which interact via a relay oscillator. The main result of our study is that for the model with type II Hodgkin–Huxley neurons, synchronization is easier to achieve than for type I Mirollo–Strogatz neurons. Synapses with short rise times (typically less than 2 ms) are more suitable to achieve zero-lag synchronization than synapses with longer rise times. With STDP the network converges to zero-lag synchronization at a faster rate and for a larger range of synaptic strengths and time delays than without STDP. However, when the delay times between the two synchronizing oscillators and the relay oscillator are different, zero phase lag may easily get lost.

The network used in this study is a simplified model for interacting neuronal populations. This obviously raises the question whether our results about zero-lag synchrony may be biased by the simplifications inherent in our model. We will argue in the next paragraphs below that this is not the case. Our choice of indirect interactions between oscillating neuronal populations, i.e., via a relay oscillator, was inspired by previous studies, which showed that pulse-coupled neuronal oscillators with direct excitatory coupling and signal delays in general do not oscillate at zero phase lag (van Vreeswijk et al., 1994; Ernst et al., 1995, 1998; Knoblauch and Sommer, 2003; Zeitler et al., 2009), unless the neurons are of type II with biphasic PRC’s (Goel and Ermentrout, 2002; Woodman and Canavier, 2011). Inhibitory coupling between directly interacting oscillators can cause near zero-lag synchrony (van Vreeswijk et al., 1994; Zeitler et al., 2009). However, the dominant connectivity between cortical areas, such as V1, V2, V4, and FEF, is excitatory, rather than inhibitory. These considerations led Fischer et al. (2006) and Vicente et al. (2008) to postulate a network model of oscillators interacting via a relay oscillator, which supports zero-lag synchrony. Subcortical structures like the thalamus are good physiological candidates for such a mediating relay (Theyel et al., 2010). Our study elaborates on this relay network model.

The most simplified version of our model assumes that the oscillators used to represent neuronal population activity are of the Mirollo–Strogatz type. The Mirollo–Strogatz oscillator corresponds to the type I neuron class (Izhikevich, 2007). Although there is evidence that cortical pyramidal cells can switch between type I and type II by means of cholinergic modulation (Ermentrout et al., 2001; Jeong and Gutkin, 2007; Prescott et al., 2008; Stiefel et al., 2008, 2009), the majority of pyramidal cells in neocortex seems to be type I neurons (Reyes and Fetz, 1993a,b; Kawaguchi, 1995; Erisir et al., 1999; Tateno et al., 2004). Our results show that synchronization is hard to achieve for the model with type I MS neurons for weak and strong synaptic coupling strengths, unless the delays are relatively long. In order to appreciate this result, one should realize that the literature on this topic is divided in studies assuming weak coupling using infinitesimal Phase Response Curves and others assuming strong synaptic coupling. Our results are in agreement with the results of Ermentrout (1996), who used a perturbation method, which is equivalent to assuming weak coupling, for networks of type I neurons with excitatory coupling. However, Mirollo and Strogatz (1990) reported that for almost all initial conditions, a network with strongly coupled type I neurons (without delays!) evolves to a state with synchronous firing. When delays are involved, zero-lag synchrony is lost (Ernst et al., 1995, 1998). Recently, Wang et al. (2012) showed that synchrony in a network with strongly coupled type I oscillators is possible in the absence of delays or with delays greater than half the network period. Although we did not study the model with delays exceeding 0.5, our results reveal the largest amount of synchrony for long delays, which is in agreement with the results by Wang et al. (2012).

Replacing the Mirollo–Strogatz neurons by more realistic type II Hodgkin–Huxley neurons allows for a broader range of synaptic strengths and time delays that is compatible with zero-lag synchronization. This result suggests a functional role for changes in neuronal properties from type I to type II, in agreement with suggestions by Prescott et al. (2008). However, there are no experimental data available yet which can be used to test the hypothesis that the properties of pyramidal cells change from type I to type II when synchrony arises in neuronal populations, as far as we know. Another possibility might be that properties of pyramidal cells do not change from type I to type II, but that the activity of interneurons causes strong inhibition after firing of the pyramidal cells (see Börgers et al., 2010, for a more extended description of the effect of inhibition). In that case, the inhibition by the interneurons after firing causes effectively a biphasic PRC with phase delays early in the firing cycle and phase advances later in the cycle for the pyramidal cell/interneuron couple, which contributes to synchrony both for direct coupling between two pyramidal cells, as well as for a pyramidal cells interacting via a relay neuron.

Delays in networks of interacting neurons can give a variety of complex behaviors with a wealth of bifurcations and a rich phase diagram, which includes oscillatory bumps, traveling waves, lurching waves, standing waves arising via a period-doubling bifurcation, aperiodic regimes, and regimes of multistability (Roxin et al., 2005). Synchronous firing is just one of these modes, which only occurs for a limited range of model parameters. A neuronal property, which greatly contributes to synchrony is that the phase response curve of the neurons is biphasic with phase delays early in the firing cycle and phase advances later in the cycle, like for type II Hodgkin–Huxley neurons. This applies both to weakly coupled oscillators (see e.g., Hansel and Mato, 1993; Hansel et al., 1993a,b) as well as for strongly coupled ones (Bressloff and Coombes, 2000; Izhikevich, 2007). Their results are in agreement with the results in our study, which show that the model with type II Hodgkin–Huxley neurons more easily leads to zero-lag synchronization than with type I MS neurons.

Another assumption of this study, which requires some more discussion, is that all oscillators in the model have identical intrinsic properties with the same oscillation period. If the intrinsic periods of the outer oscillators differ, zero-lag synchrony may get lost. Whether or not synchrony will be lost depends on the neuron type. If the synaptic input to the outer neurons, which have different natural frequencies, resets their oscillation periods to the same value, zero-lag synchronization is easily obtained. Synchronization of non-linear oscillators with different oscillatory properties is feasible if the interactions between the oscillators (the synaptic strengths in our study) are sufficiently strong (Pikovsky et al., 2001). A special condition is the situation where the intrinsic period of the relay oscillator is different from that of the outer oscillators. This might not be unusual if the relay oscillators are thalamic cells and the outer oscillators cortical cells. In this case, the synchronization properties change quantitatively but not qualitatively. These results have been confirmed by simulations (not shown) but can be understood from the following: If we make the period of the relay oscillator different from that of the outer oscillators, the combinations of synaptic strength and delay where input can elicit spikes immediately after arrival change only slightly with adjustments of the period. Hence the boundaries of regions with driven, pacemaker, and slave synchrony will change quantitatively, but not qualitatively, unless the differences in the period become too large.

If the periods of the two outer oscillators are different from each other and if input from the relay oscillator does not make the period of the outer oscillators the same, input from the relay oscillator to the outer neurons will elicit spikes at different times. In that case, the spike input from the outer oscillators to the relay neuron also arrives at different times. This is essentially equivalent to the situation with different delay times, which we have studied (see When Delay Times from the Relay Neuron to Outer Neurons are not identical), where we have shown that zero-lag synchrony is easily lost if the delay times become different (see also Figure 10). Therefore, synaptic coupling strengths should be sufficiently strong to ensure zero-lag synchrony when the oscillation periods of the outer oscillators differ.

In our study we have introduced “SQ” as a measure for the robustness of synchrony against variations in the initial phases of the oscillators. SQ was used together with the “CP” to assess zero-lag synchrony between the two outer oscillators in our relay network. The time interval for “synchrony” was chosen as spike coincidence within 0.5 ms (Engel and Singer, 2001), which in experimental settings is long enough to take into account typical noise on spike-timings but short enough to speak about “zero-lag.” Increasing the time interval to 1 ms did not affect the results qualitatively, though quantitatively some minor differences were observed.

In agreement with Knoblauch and Sommer (2003), we found that if STDP adapts synaptic coupling, the network state converges more easily to a stable state with zero-lag synchrony (see Figures 6 and 9). However, adaptation of the synapses by STDP often took quite some time [in general more than 500 cycles (one session corresponds to 60 cycles), see Figure 7], which implies that STDP may not always play a dominant role for the rapid development of zero-lag synchronization. Vicente et al. (2008) reported that the mechanism of synchronization rests on the ability of an excitatory postsynaptic potential to modify the firing latencies of a postsynaptic neuron in a consistent manner. We agree with this conclusion, but our results show that the mechanism of STDP may take too much time (considerably more than the observed time range of 200–250 ms in visual perception, see Rodriguez et al. (1999)), to generate zero-lag synchrony for oscillations in the gamma frequency range.

Overall, our results demonstrate that gamma oscillations in various cortical areas can be synchronized at zero-lag in a network model where neuronal oscillators are coupled via a relay oscillator, in agreement with previous studies (Fischer et al., 2006; Vicente et al., 2008). In addition we show that STDP expands the range of parameter values, which allow zero-lag synchrony.

## Conflict of Interest Statement

The authors declare that the research was conducted in the absence of any commercial or financial relationships that could be construed as a potential conflict of interest.

## Figures and Tables

**Figure A1:**
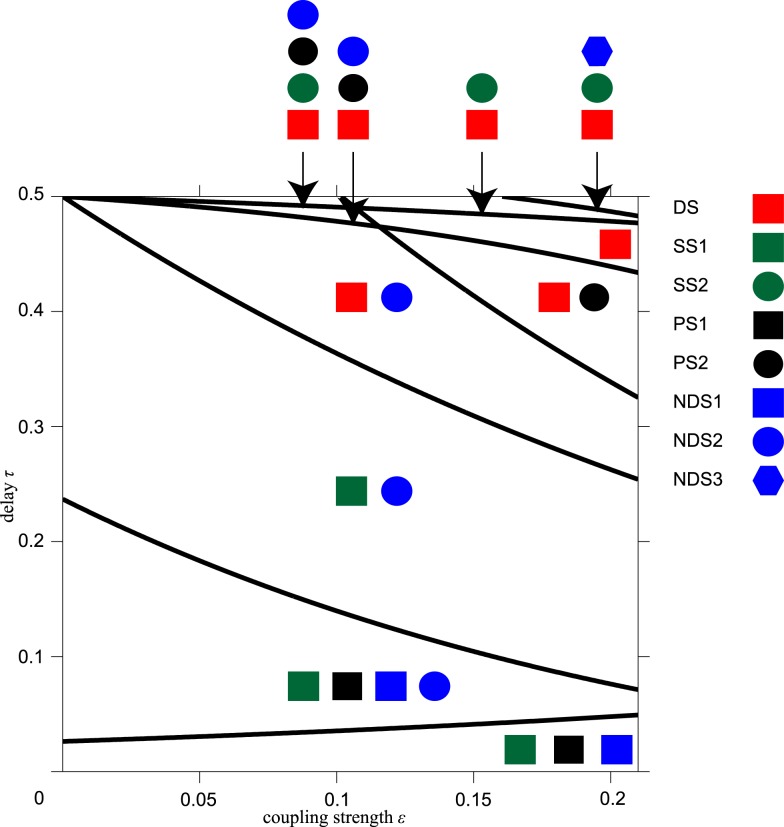
**Synchronization regions in the parameter plane of delay time and coupling strength**. Different combinations of possible synchrony modes divide the parameter plane of delay time τ and synaptic weight ε into ten distinct regions. These are shown separated by black lines and the corresponding synchrony modes are indicated by symbols referring to results listed in the Appendix: driven synchrony – Eq. A7 “DS” in Appendix; pacemaker synchrony – Eq. A10 “PS1” and “PS2” in Appendix; slave synchrony – Eq. A12 “SS1” and “SS2” in Appendix; and non-driven synchrony – Eq. A13 “NDS1,” “NDS2,” and “NDS3” in Appendix.

## Appendix

### Detailed analysis of phase-locking and stability

Analytical derivations of the various synchrony modes and their stability are provided. An overview of these results is provided by Figure A1.

#### Synchrony modes derived from the phase-locking equation

To investigate the existence of zero-lag synchrony as a function of τ and ε, we will derive the 1:1 synchronized phase-locking equation of the oscillators, cf. Figure 1B. The firing of the relay neuron $t_{2,n-1}^{\text{spike}}$ is chosen as reference time, i.e., $t_{2,n-1}^{\text{spike}}=0.$ For zero-lag synchrony there exists some value þeta ∈ [0, 1) such that the firing times $t_{1,n-1}^{\text{spike}}$ and $t_{3,n-1}^{\text{spike}}$ of both neurons 1 and 3 are equal to θ*T*, and $t_{1,n}^{\text{spike}}$ and $t_{3,n}^{\text{spike}}$ are equal to θ*T* + *T*, with *T* the period of the oscillations in the network. The spikes from the outer oscillators arrive at the relay oscillator at phase τ + θ*T*/*T*_0_, if τ ≤ (1 − θ)*T*/*T*_0_, or at τ − (1 − θ)*T*/*T*_0_, if τ > (1 − θ)*T*/*T*_0_. The new period of the inner oscillator 2 is hence given by

(A1) $$T=\left\{\begin{array}{cc} T_{\text{0}}-T_{\text{0}}\Delta \;\;\left(\left.\tau +\theta \frac{T}{T_{\text{0}}}\right|2\varepsilon \right)\; & \text{for }\tau \leq \left(\text{1 - }\theta \right)\frac{T}{T_{\text{0}}}\\ T_{\text{0}}-T_{\text{0}}\Delta \;\left(\left.\tau -\left(1-\theta \right)\frac{T}{T_{\text{0}}}\right|2\varepsilon \right)\; & \text{for }\tau >\left(\text{1 - }\theta \right)\frac{T}{T_{\text{0}}}\\ \; \end{array}\right.,$$

where Δφ is defined in Eqs 6 and 7 of the main text, repeated here for convenience:

$$\begin{array}{llll} \Delta \varphi_{i}\left(\varphi_{i}\;\left|\;\varepsilon_{ij}\right.\right) & =\left\{\begin{array}{cc} \chi_{b}\left(\varepsilon_{ij}\right)+\beta_{b}\left(\varepsilon_{ij}\right)\varphi_{i}\; & \text{for}\;0\leq \varphi_{i}<\varphi_{c}\\ 1-\varphi_{i}\; & \text{for}\;\varphi_{c}\leq \varphi_{i}<1\\ \; \end{array}\right., & & \text{(A2)}\\ \chi_{b}\left(x\right) & \equiv \frac{\beta_{b}\left(x\right)}{\beta_{b}\left(1\right)},\;\beta_{b}\left(x\right)\equiv e^{bx}-1. & & \text{(A3)} \end{array}$$

The spike from oscillator 2 will arrive at oscillators 1 and 3 at phase τ + (1 − θ)*T*/*T*_0_, if τ ≤ θ*T*/*T*_0_, or τ − θ*T*/*T*_0_, if τ > θ*T*/*T*_0_. The new period of the outer oscillators 1 and 3 is hence given by

(A4) $$T=\left\{\begin{array}{cc} T_{\text{0}}-T_{\text{0}}\Delta \varphi \left(\left.\tau +\left(1-\theta \right)\frac{T}{T_{\text{0}}}\right|\varepsilon \right)\; & \text{for }\tau \leq \theta \frac{T}{T_{\text{0}}}\\ T_{\text{0}}-T_{\text{0}}\Delta \varphi \left(\left.\tau -\theta \frac{T}{T_{\text{0}}}\right|\varepsilon \right)\; & \text{for }\tau >\theta \frac{T}{T_{\text{0}}}\\ \; \end{array}\right..$$

Equations A1 and A4 are the 1:1 phase-locking equations, which give a relation between the synaptic weight ε, the conduction delay τ, and the new period of the oscillators *T*.

Using Eqs A1 and A4, we will derive four synchronization modes: driven, pacemaker, slave, and non-driven synchrony. In driven synchrony spikes from the outer neurons immediately initiate a spike of the relay neuron, and vice versa, cf. top of Figure 2B. In pacemaker synchrony arrival of a spike from the relay oscillator at the outer oscillators immediately elicits a spike, but not vice versa, cf. bottom of Figure 2B. When the relay neuron spikes immediately after spikes from the outer neurons, but not vice versa, we call this slave synchrony, cf. middle of Figure 2B. Finally, non-driven synchrony occurs when neither the relay neuron nor the outer neurons spike immediately after input.

First, we consider driven synchrony. In order for all input spikes to elicit a spike in the receiving oscillators, we must have the following: for the inner oscillator φ*_c_*(2ε) ≤ τ + θ*T*/*T*_0_ if τ ≤ (1 − θ)*T*/*T*_0_, and φ*_c_*(2ε) ≤ τ − (1 − θ)*T*/*T*_0_ if τ > (1 − θ)*T*/*T*_0_; and for the outer oscillators φ*_c_*(ε) ≤ τ + (1 − θ)*T*/*T*_0_, if τ ≤ θ*T*/*T*_0_, and φ*_c_*(ε) ≤ τ − θ*T*/*T*_0_, if τ > θ*T*/*T*_0_. Using Eqs A1, A2, and A4, we can then rewrite the new period of the inner oscillator as

(A5) $$T=\left\{\begin{array}{cc} \tau T_{\text{0}}+\theta T\; & \text{for }\tau \leq \left(1-\theta \right)\frac{T}{T_{\text{0}}}\text{ and }\varphi_{c}\left(2\varepsilon \right)\leq \tau +\theta \frac{T}{T_{\text{0}}}\\ \tau T_{\text{0}}-\left(1-\theta \right)T\; & \text{for }\tau >\left(1-\theta \right)\frac{T}{T_{\text{0}}}\text{ and }\varphi_{c}\left(2\varepsilon \right)\leq \tau -\left(1-\theta \right)\frac{T}{T_{\text{0}}}\\ \; \end{array}\right.,$$

and the new period of the outer oscillators as

(A6) $$T=\left\{\begin{array}{cc} \tau T_{\text{0}}+\left(1-\theta \right)T\; & \text{for }\tau \leq \theta \frac{T}{T_{\text{0}}}\text{ and }\varphi_{c}\left(\varepsilon \right)\leq \tau +\left(1-\theta \right)\frac{T}{T_{\text{0}}}\\ \tau T_{\text{0}}-\theta T\; & \text{for }\tau >\theta \frac{T}{T_{\text{0}}}\text{ and }\varphi_{c}\left(\varepsilon \right)\leq \tau -\theta \frac{T}{T_{\text{0}}}\\ \; \end{array}\right..$$

We can now solve for θ and *T* by using one part of Eqs A5 and A6 each, and combining them under the condition that the new periods *T* of the inner and outer oscillators, respectively, must be identical to achieve synchrony. This yields four possible combinations:

(A7) $$\left(\theta ,\frac{T}{T_{\text{0}}}\right)=\left\{\begin{array}{cc} \left(\frac{1}{2},2\tau \right)\; & \text{for }\varphi_{c}\left(\varepsilon \right)\leq 2\tau \text{ and }\varphi_{c}\left(2\varepsilon \right)\leq 2\tau \;\text{DS}\\ \left(0,\tau \right)\; & \text{for }\varphi_{c}\left(\varepsilon \right)\leq \tau \text{ and }\varphi_{c}\left(2\varepsilon \right)\leq \tau \;\\ \left(1,\tau \right)\; & \text{for }\varphi_{c}\left(\varepsilon \right)\leq \tau \text{ and }\varphi_{c}\left(2\varepsilon \right)\leq \tau \\ \left(\frac{1}{2},\frac{2}{\text{3}}\tau \right)\; & \text{for }\varphi_{c}\left(\varepsilon \right)\leq \frac{2}{\text{3}}\tau \text{ and }\varphi_{c}\left(2\varepsilon \right)\leq \frac{2}{\text{3}}\tau \\ \; \end{array}\right..$$

For biologically realistic conditions τ ∈ (0, 0.5) and ε ∈ (0, 0.21) and our choice *b* = 3, only the driven synchrony mode labeled “DS” turns out to be valid. The firing dynamics of “DS” is illustrated in the top of Figure 2B. For the other cases the φ*_c_* conditions are not fulfilled and therefore these solutions are rejected. Moreover, the third solution also has a θ value out of range for 1:1 phase-locking. We will similarly identify the valid modes of other solutions below. Since φ*_c_*(2ε) ≤ φ*_c_*(ε), φ*_c_*(ε) ≤ 2τ implies φ*_c_*(2ε) ≤ 2τ and the φ*_c_*(ε) ≤ 2τ constraint is sufficient. This constraint divides the ε and τ parameter space into two regions: driven synchrony is possible in the region where φ*_c_*(ε) ≤ 2τ but not in the region where φ*_c_*(ε) > 2τ. These two regions are separated by the line φ*_c_*(ε) = 2τ illustrated as the line separating regions I and II in Figures 2A,C.

Second, we investigate pacemaker synchrony. For pacemaker synchrony only the outer oscillators spike directly upon input, but not the inner one. Thus for the inner oscillator, we must have φ*_c_*(2ε) > τ + θ*T*/*T*_0_ if τ ≤ (1 − θ)*T*/*T*_0_, and φ*_c_*(2ε) > τ − (1 − θ)*T*/*T*_0_ if τ > (1 − θ)*T*/*T*_0_. For the outer oscillators, we must have φ*_c_*(ε) ≤ τ + (1 − θ)*T*/*T*_0_ if τ ≤ θ*T*/*T*_0_, and φ*_c_*(ε) ≤ τ − θ*T*/*T*_0_ if τ > θ*T*/*T*_0_. Using Eq. A2 in Appendix, we can rewrite the new period of the inner oscillator given by Eq. A1 in Appendix as

$$\begin{array}{cc} T=\left\{\begin{array}{cc} T_{\text{0}}\gamma_{b}\left(2\varepsilon \right)-\theta T\beta_{b}\left(2\varepsilon \right)\; & \text{for }\tau \leq \left(1-\theta \right)\frac{T}{T_{\text{0}}}\text{ and }\varphi_{c}\left(2\varepsilon \right)>\tau +\theta \frac{T}{T_{\text{0}}}\\ T_{\text{0}}\gamma_{b}\left(2\varepsilon \right)+\left(1-\theta \right)T\beta_{b}\left(2\varepsilon \right)\; & \text{for }\tau >\left(1-\theta \right)\frac{T}{T_{\text{0}}}\text{ and }\varphi_{c}\left(2\varepsilon \right)>\tau -\left(1-\theta \right)\frac{T}{T_{\text{0}}}\\ \; \end{array}\right., & \text{(A8)}\\ \gamma_{b}\left(x\right)\equiv 1-\chi_{b}\left(x\right)-\beta_{b}\left(x\right)\tau , & \text{(A9)} \end{array}$$

where as the new period of the outer oscillators is given by Eq. A6 in Appendix. Solving for θ and *T* as before, we find the following four possibilities:

(A10) $$\left(\theta ,\frac{T}{T_{\text{0}}}\right)=\left\{\begin{array}{cc} \left(\tau \frac{T_{\text{0}}}{T},\gamma_{b}\left(2\varepsilon \right)-\beta_{b}\left(2\varepsilon \right)\tau \right)\; & \text{for }\varphi_{c}\left(\varepsilon \right)\leq \frac{T}{T_{\text{0}}}\text{ and }\varphi_{c}\left(2\varepsilon \right)>2\tau \;\text{PS1}\\ \left(\tau \frac{T_{\text{0}}}{T}-1,\frac{\gamma_{b}\left(2\varepsilon \right)-\beta_{b}\left(2\varepsilon \right)\tau }{1-\beta_{b}\left(2\varepsilon \right)}\right)\; & \text{for }\varphi_{c}\left(\varepsilon \right)\leq \frac{T}{T_{\text{0}}}\text{ and }\varphi_{c}\left(2\varepsilon \right)>2\tau -\frac{T}{T_{\text{0}}}\\ \left(\tau \frac{T_{\text{0}}}{T},\frac{\gamma_{b}\left(2\varepsilon \right)-\beta_{b}\left(2\varepsilon \right)\tau }{1-\beta_{b}\left(2\varepsilon \right)}\right)\; & \text{for }\varphi_{c}\left(\varepsilon \right)\leq \frac{T}{T_{\text{0}}}\text{ and }\varphi_{c}\left(2\varepsilon \right)>2\tau -\frac{T}{T_{\text{0}}}\;\text{PS2}\\ \left(\tau \frac{T_{\text{0}}}{T}-1,\frac{\gamma_{b}\left(2\varepsilon \right)-\beta_{b}\left(2\varepsilon \right)\tau }{1-2\beta_{b}\left(2\varepsilon \right)}\right)\; & \text{for }\varphi_{c}\left(\varepsilon \right)\leq \frac{T}{T_{\text{0}}}\text{ and }\varphi_{c}\left(2\varepsilon \right)>2\tau -2\frac{T}{T_{\text{0}}}\\ \; \end{array}\right..$$

The two valid modes for pacemaker synchrony given our constraints are labeled “PS1” and “PS2” in Eq. A10 in Appendix, respectively. Within our ranges of interest for ε and τ, one can check numerically for the “PS1” and “PS2” pacemaker synchrony that φ*_c_*(ε) ≤ *T*/*T*_0_ implies φ*_c_*(2ε) > 2τ and φ*_c_*(2ε) > 2τ − *T*/*T*_0_, respectively. Hence, only the φ*_c_*(ε) ≤ *T*/*T*_0_ constraint is required. “PS1” pacemaker synchrony is possible in the region {(ε,τ) ∈ [0, 0.21] × [0, 0.5]|φ*_c_*(ε) ≤ *T*/*T*_0_}; while “PS2” is possible in the region {(ε,τ) ∈ [0, 0.21] × [0, 0.5]|φ*_c_*(ε) ≤ *T*/*T*_0_ and (1 − θ)*T*/*T*_0_ < τ}, where (1 − θ)*T*/*T*_0_ < τ refers to the condition given in Eq. A1 in Appendix. The equality φ*_c_*(ε) = *T*/*T*_0_ of “PS1” gives the line separating regions II and III in Figures 2A,C. The firing dynamics for “PS1” pacemaker synchrony is illustrated in Figure 2B.

Third, in slave synchrony only the inner oscillator spikes upon input, i.e., in case of the inner oscillator, we must have φ*_c_*(2ε) ≤ τ + θ*T*/*T*_0_ if τ ≤ (1 − θ)*T*/*T*_0_, and φ*_c_*(2ε) ≤ τ − (1 − θ)*T*/*T*_0_ if τ > (1 − θ)*T*/*T*_0_. For the outer oscillators, we must have φ*_c_*(ε) > τ + (1 − θ)*T*/*T*_0_ if τ ≤ θ*T*/*T*_0_, and φ*_c_*(ε) > τ − θ*T*/*T*_0_ if τ > θ*T*/*T*_0_. The new period of the inner oscillator is given by Eq. A5 in Appendix and from Eqs A2 and A4 we obtain the following expression for the new period of the outer oscillator

(A11) $$T=\left\{\begin{array}{cc} T_{\text{0}}\gamma_{b}\left(\varepsilon \right)-\left(1-\theta \right)T\beta_{b}\left(\varepsilon \right)\; & \text{for }\tau \leq \theta \frac{T}{T_{\text{0}}}\;\text{ and }\varphi_{c}\left(\varepsilon \right)>\tau +\left(1-\theta \right)\frac{T}{T_{\text{0}}}\\ T_{\text{0}}\gamma_{b}\left(\varepsilon \right)+\theta T\beta_{b}\left(\varepsilon \right)\; & \text{for }\tau >\theta \frac{T}{T_{\text{0}}}\;\text{ and }\varphi_{c}\left(\varepsilon \right)>\tau -\theta \frac{T}{T_{\text{0}}}\\ \; \end{array}\right..$$

The four combinations are then:

(A12) $$\left(\theta ,\frac{T}{T_{\text{0}}}\right)=\left\{\begin{array}{cc} \left(1-\tau \frac{T_{\text{0}}}{T},\gamma_{b}\left(\varepsilon \right)-\beta_{b}\left(\varepsilon \right)\tau \right)\; & \text{for }\varphi_{c}\left(\varepsilon \right)>2\tau \text{ and }\varphi_{c}\left(2\varepsilon \right)\leq \frac{T}{T_{\text{0}}}\;\text{SS1}\\ \left(1-\tau \frac{T_{\text{0}}}{T},\frac{\gamma_{b}\left(\varepsilon \right)-\beta_{b}\left(\varepsilon \right)\tau }{1-\beta_{b}\left(\varepsilon \right)}\right)\; & \text{for }\varphi_{c}\left(\varepsilon \right)>2\tau -\frac{T}{T_{\text{0}}}\text{ and }\varphi_{c}\left(2\varepsilon \right)\leq \frac{T}{T_{\text{0}}}\;\text{SS2}\\ \left(2-\tau \frac{T_{\text{0}}}{T},\frac{\gamma_{b}\left(\varepsilon \right)-\beta_{b}\left(\varepsilon \right)\tau }{1-\beta_{b}\left(\varepsilon \right)}\right)\; & \text{for }\varphi_{c}\left(\varepsilon \right)>2\tau -\frac{T}{T_{\text{0}}}\text{ and }\varphi_{c}\left(2\varepsilon \right)\leq \frac{T}{T_{\text{0}}}\\ \left(2-\tau \frac{T_{\text{0}}}{T},\frac{\gamma_{b}\left(\varepsilon \right)-\beta_{b}\left(\varepsilon \right)\tau }{1-2\beta_{b}\left(\varepsilon \right)}\right)\; & \text{for }\varphi_{c}\left(\varepsilon \right)>2\tau -2\frac{\text{T}}{{\text{T}}_{\text{0}}}\text{ and }\varphi_{c}\left(2\varepsilon \right)\leq \frac{\text{T}}{{\text{T}}_{\text{0}}}\\ \; \end{array}\right..$$

The two valid slave synchronization modes will be called “SS1” and “SS2,” respectively. Within our ranges of interest for ε and τ, one can check numerically for the “SS1” slave synchrony mode that φ*_c_*(ε) > 2τ implies φ*_c_*(2ε) ≤ *T*/*T*_0_; whereas the constraints for “SS2” are always fulfilled in our ranges for ε and τ. Hence “SS1” slave synchrony, the firing dynamic of which is illustrated in Figure 2B, is possible in the region {(ε,τ) ∈ [0, 0.21] × [0, 0.5]|φ*_c_*(ε) > 2τ}; while “SS2” is possible in the region {(ε,τ) ∈ [0, 0.21] × [0, 0.5]|τ > θ*T*/*T*_0_}, where τ > θ*T*/*T*_0_ refers to the condition given in Eq. A4 in Appendix. The equality τ = θ*T*/*T*_0_ of “SS2” gives the line separating regions I and IV in Figures 2A,C.

Finally, for non-driven synchrony none of oscillators fires immediately after receiving a spike. This implies that for the inner oscillator φ*_c_*(2ε) > τ + θ*T*/*T*_0_ if τ ≤ (1 − θ)*T*/*T*_0_, and φ*_c_*(2ε) > τ − (1 − θ)*T*/*T*_0_ if τ > (1 − θ)*T*/*T*_0_. For the outer oscillators φ*_c_*(ε) > τ + (1 − θ)*T*/*T*_0_ if τ ≤ θ*T*/*T*_0_, and φ*_c_*(ε) > τ − θ*T*/*T*_0_ if τ > θ*T*/*T*_0_. The new period is then given by Eqs A8 and A11 for the inner and outer oscillators, respectively. The resulting combinations are:

(A13) $$\left(\theta ,\frac{T}{T_{\text{0}}}\right)=\left\{\begin{array}{cc} \left(\frac{\rho_{++-0}}{\rho_{0+0+}},\frac{\rho_{0+0+}}{\zeta_{+}}\right)\; & \text{for }\varphi_{c}\left(\varepsilon \right)>\tau -\frac{\rho_{+0--}}{\zeta_{+}}\text{ and }\varphi_{c}\left(2\varepsilon \right)>\tau +\frac{\rho_{++-0}}{\zeta_{+}}\;\text{NDS1}\\ \left(\frac{\rho_{+0-0}}{\rho_{0+0+}},\frac{\rho_{0+0+}}{\zeta_{0}}\right)\; & \text{for }\varphi_{c}\left(\varepsilon \right)>\tau -\frac{\rho_{+0-0}}{\zeta_{0}}\text{ and }\varphi_{c}\left(2\varepsilon \right)>\tau +\frac{\rho_{+0-0}}{\zeta_{0}}\\ \left(\frac{\rho_{++-+}}{\rho_{0+0+}},\frac{\rho_{0+0+}}{\zeta_{0}}\right)\; & \text{for }\varphi_{c}\left(\varepsilon \right)>\tau -\frac{\rho_{+0-0}}{\zeta_{0}}\text{ and }\varphi_{c}\left(2\varepsilon \right)>\tau +\frac{\rho_{+0-0}}{\zeta_{0}}\;\text{NDS2}\\ \left(\frac{\rho_{+0-+}}{\rho_{0+0+}},\frac{\rho_{0+0+}}{\zeta_{-}}\right)\; & \text{for }\varphi_{c}\left(\varepsilon \right)>\tau -\frac{\rho_{+0-+}}{\zeta_{-}}\text{ and }\varphi_{c}\left(2\varepsilon \right)>\tau +\frac{\rho_{+--0}}{\zeta_{-}}\;\text{NDS3} \end{array}\right.$$

with ρ_mnop_ = [*m* + *n*β*_b_*(ε)]γ*_b_*(2ε) + [*o* + *p*β*_b_*(2ε)]γ*_b_*(ε), where indices can take on the values + 1, 0, −1 as represented by the signs, and likewise ζ*_m_* ≡ β*_b_*(ε) + β*_b_*(2ε) + *m*β*_b_*(ε)β*_b_*(2ε). The three valid non-driven synchronization modes will be called NDS1, NDS2, and NDS3, respectively. From the constraints of ε and τ for “NDS1” slave synchrony, φ*_c_*(ε) > τ − ρ_+0−−_/ζ_+_ implies φ*_c_*(2ε) > τ + ρ_++−0_/ζ_+_. For the “NDS2” slave synchrony, φ*_c_*(ε) > τ − ρ_+0−0_/ζ_0_ implies φ*_c_*(2ε) > τ + ρ_+0−0_/ζ_0_. But the constraints of “NDS3” fulfill automatically in our range of ε and τ. Hence the “NDS1” slave synchrony is possible in the region {(ε,τ)∈[0, 0.21] × [0, 0.5]|φ*_c_*(ε) > τ − ρ_+0−−_/ζ_+_}; while “NDS2” is possible in the region {(ε,τ) ∈ [0, 0.21] × [0, 0.5]|φ*_c_*(ε) > τ − ρ_+0−0_/ζ_0_ and τ > (1 − θ)*T*/*T*_0_} and “NDS3” in the region {(ε,τ) ∈ [0, 0.21] × [0, 0.5]|τ > (1 − θ)*T*/*T*_0_}, where τ > (1 − θ)*T*/*T*_0_ refers to the condition given in Eq. A1 in Appendix.

Based on different combinations of the synchronization modes DS from Eq. A7 in Appendix, PS1 and PS2 from Eq. A10 in Appendix, SS1 and SS2 from Eq. A12 in Appendix, and NDS1, NDS2, and NDS3 from Eq. A13 in Appendix, we can separate the parameter plane of time delay τ and synaptic weight ε into ten regions, see Figure A1. In summary, the regions in the ε and τ parameter space, where the modes are possible, are given by

(A14) $$\begin{array}{cc} \left\{\left(\varepsilon ,\tau \right)\in \left[0,0.21\right]\times \left[0,0.5\right]|\varphi_{c}\left(\varepsilon \right)\leq 2\tau \right\} & \text{for DS},\\ \left\{\left(\varepsilon ,\tau \right)\in \left[0,0.21\right]\times \left[0,0.5\right]|\varphi_{c}\left(\varepsilon \right)\leq T/T_{0}\right\} & \text{for PS1},\\ \left\{\left(\varepsilon ,\tau \right)\in \left[0,0.21\right]\times \left[0,0.5\right]|\varphi_{c}\left(\varepsilon \right)\leq T/T_{0}\text{ and }\left(1-\theta \right)T/T_{0}<\tau \right\} & \text{for PS2},\\ \left\{\left(\varepsilon ,\tau \right)\in \left[0,0.21\right]\times \left[0,0.5\right]|\varphi_{c}\left(\varepsilon \right)>2\tau \right\} & \text{for SS1},\\ \left\{\left(\varepsilon ,\tau \right)\in \left[0,0.21\right]\times \left[0,0.5\right]|\tau >\theta T/T_{0}\right\} & \text{for SS2},\\ \left\{\left(\varepsilon ,\tau \right)\in \left[0,0.21\right]\times \left[0,0.5\right]|\varphi_{c}\left(\varepsilon \right)>\tau -\rho_{+0--}/\zeta_{+}\right\} & \text{for NDS1},\\ \left\{\left(\varepsilon ,\tau \right)\in \left[0,0.21\right]\times \left[0,0.5\right]|\varphi_{c}\left(\varepsilon \right)>\tau -\rho_{+0-0}/\zeta_{0}\text{ and }\tau >\left(1-\theta \right)T/T_{0}\right\} & \text{for NDS2},\\ \left\{\left(\varepsilon ,\tau \right)\in \left[0,0.21\right]\times \left[0,0.5\right]|\tau >\left(1-\theta \right)T/T_{0}\right\} & \text{for NDS3}. \end{array}$$

#### Stability analysis of synchrony modes

In order to investigate the stability of driven synchrony, we assume that the fixed point Eq. A7 “DS” in Appendix suffers from small phase perturbations (φ_1_, φ_3_) = (τ + δφ_1_, τ + δφ_1_) at time $t_{2,n-1}^{\text{spike}}.$ Driven synchrony is asymptotically stable, if there is a δ > 0 such that the phases of the outer oscillators will be closer to τ at the next spike time $t_{2,n}^{\text{spike}}$ for $\sqrt{\delta \varphi_{1}^{2}+\delta \varphi_{3}^{2}}<\delta .$ Since φ*_c_*(ε) < 2τ, we can define δ ≡ 2τ − φ*_c_*(ε) > 0, and by this definition φ*_c_*(ε) ≡ 2τ − δ. The spike from the relay arrives when oscillator 1 has phase 2τ − δ < φ_1_ < 2τ + δ, which exceeds the critical phase φ*_c_*(ε). Therefore, oscillator 1 will spike immediately after input. Since the same argument applies to oscillator 3, the outer oscillators will again spike in synchrony, proving asymptotic stability for small perturbations. Similar arguments can be made to show that the pacemaker synchrony of Eq. A10 “PS1” and “PS2” in Appendix is asymptotically stable.

Next we investigate the stability of the slave synchronization mode Eq. A12 “SS1” in Appendix. Here one has to calculate the eigenvalues λ of the Jacobian of the return map near the fixed point of the outer oscillator phases (Zeitler et al., 2009). If all eigenvalues are within the unit circle (|λ| < 1), then the fixed point is asymptotically stable, otherwise it is unstable. Assume at time $t_{2,n-1}^{\text{spike}}$ that phases are perturbed away from the fixed point of Eq. A12 “SS1” in Appendix: (φ_1_, φ_3_) = (τ + δφ_1_, τ + δφ_3_). Thus synaptic inputs from oscillators 1 and 3 will arrive at oscillator 2 at slightly different times. Without loss of generality, we assume that the synaptic input from oscillator 1 arrives earlier. This first synaptic input can make the relay oscillator spike immediately, if φ*_c_*(ε) < τ + θ*T*/*T*_0_. Assuming that the perturbation is small enough for the solution in Eq. A12 “SS1” in Appendix to remain applicable, we find φ*_c_*(ε) < τ + θ*T*/*T*_0_ = 1 − χ*_b_*(ε) − 2β*_b_*(ε)τ = [1 + β*_b_*(ε)]φ*_c_*(ε) − 2β*_b_*(ε)τ. For non-zero coupling this is equivalent to 2τ < φ*_c_*(ε), which is biologically reasonable (Tsubo et al., 2007). Therefore we assume φ*_c_*(ε) < τ + θ*T*/*T*_0_, and the spike from oscillator 1 will make the relay oscillator spike instantaneously when it arrives. Define ${\tilde{\varphi }}_{1}$ as the phase of oscillator 1 at this relay spike time $t_{2,n}^{\text{spike}},$ then obviously ${\tilde{\varphi }}_{1}=\tau ,$ the conduction delay.

To find the phase ${\tilde{\varphi }}_{3}$ of oscillator 3 when oscillator 2 spikes at $t_{2,n}^{\text{spike}},$ we first derive the previous spike times of the outer oscillators $t_{1,n-1}^{\text{spike}}$ and $t_{3,n-1}^{\text{spike}}.$ The phase of oscillator 1 is τ + δφ_1_ when the relay oscillator fires at $t_{2,n-1}^{\text{spike}}\equiv 0.$ After a delay τ*T*_0_, the spike from the relay oscillator arrives at oscillator 1, thus at its phase $\varphi_{1}^{\text{old}}=2\tau +\delta \varphi_{1}.$ If δφ_1_ is small enough we can still assume 2τ + δφ_1_ < φ*_c_*(ε), i.e., this input from the relay will not make oscillator 1 spike instantaneously. Instead its phase will jump to $\varphi_{1}^{\text{new}}=\varphi_{1}^{\text{old}}+\chi_{b}\left(\varepsilon \right)+\beta_{b}\left(\varepsilon \right)\varphi_{1}^{\text{old}},$ cf. Eq. A2 in Appendix. After receiving the spike, it takes an amount of time $T_{\text{0}}\left(1-\varphi_{1}^{\text{new}}\right)$ until oscillator 1 spikes at $t_{1,n-1}^{\text{spike}}.$ Hence $t_{1,n-1}^{\text{spike}}$is obtained by adding the conduction delay τ*T*_0_ between oscillator 2 and 1 to the time$T_{\text{0}}\left(1-\varphi_{1}^{\text{new}}\right)$ that oscillator 1 still needs to evolve before spiking. Spike time $t_{3,n-1}^{\text{spike}}$ can be obtained in a similar way, and thus one finds:

$$\begin{array}{llll} t_{1,n-1}^{\text{spike}} & =T_{\text{0}}\left\{1-\chi_{b}\left(\varepsilon \right)-\beta_{b}\left(\varepsilon \right)\tau -\left[1+\beta_{b}\left(\varepsilon \right)\right]\left(\tau +\delta \varphi_{1}\right)\right\}, & & \text{(A15)}\\ t_{3,n-1}^{\text{spike}} & =T_{\text{0}}\left\{1-\chi_{b}\left(\varepsilon \right)-\beta_{b}\left(\varepsilon \right)\tau -\left[1+\beta_{b}\left(\varepsilon \right)\right]\left(\tau +\delta \varphi_{3}\right)\right\}. & & \text{(A16)} \end{array}$$

The next spike of the relay oscillator occurs at $t_{2,n}^{\text{spike}}=t_{1,n-1}^{\text{spike}}+\tau T_{\text{0}},$ as explained above. Since ${\tilde{\varphi }}_{3}=\left(t_{2,n}^{\text{spike}}-t_{3,n-1}^{\text{spike}}\right)/T_{0}=\tau +\left(t_{1,n-1}^{\text{spike}}-t_{3,n-1}^{\text{spike}}\right)/T_{0}$, we find the following return map for our local stability analysis

$$\begin{array}{llll} {\tilde{\varphi }}_{1} & =\tau , & & \text{(A17)}\\ {\tilde{\varphi }}_{3} & =\tau -\left[1+\beta_{b}\left(\varepsilon \right)\right]\varphi_{1}+\left[1+\beta_{b}\left(\varepsilon \right)\right]\varphi_{3}. & & \text{(A18)} \end{array}$$

The corresponding Jacobian matrix is

(A19) $$\left\{\;\begin{array}{cc} 0 & 0\\ -\left[1+\beta_{b}\left(\varepsilon \right)\right] & 1+\beta_{b}\left(\varepsilon \right) \end{array}\right\}.$$

The eigenvalues of this matrix are 0 and 1 + β*_b_*(ε) > 1. Consequently, this fixed point is not stable. Equation A12 “SS2” in Appendix can be shown to be unstable in a similar manner.

Finally, we investigate the stability of non-driven synchronization Eq. A13 “NDS1” in Appendix. We will discuss the return map near the fixed point $\left(\varphi_{\text{1}}^{*},\varphi_{\text{3}}^{*}\right)=\left(\left(1-\theta \right)\text{T},\left(1-\theta \right)T\right).$ At time $t_{2,n-1}^{\text{spike}},$ we assume a small perturbation $\left(\varphi_{1},\varphi_{3}\right)=\left(\varphi_{\text{1}}^{*}+\delta \varphi_{1},\varphi_{\text{3}}^{*}+\delta \varphi_{3}\right).$ The arguments used to derive Eqs A15 and A16 hold here, except that the perturbed fixed point is $\tau +\delta \varphi_{1}\to \varphi_{\text{1}}^{*}+\delta \varphi_{1}$ and $\tau +\delta \varphi_{3}\to \varphi_{\text{3}}^{*}+\delta \varphi_{3},$ respectively. Without loss of generality, we assume that the synaptic input from oscillator 1 arrives at oscillator 2 before that from oscillator 3. Thus when oscillator 1 spikes at $t_{1,n-1}^{\text{spike}},$ the spike input from oscillator 1 arrives at oscillator 2 at phase $t_{1,n-1}^{\text{spike}}/T_{\text{0}}+\tau$ and causes the phase of oscillator 2 to change to $\chi_{b}\left(\varepsilon \right)+\left[\text{1}+\beta_{b}\left(\varepsilon \right)\right]\left[t_{1,n-1}^{\text{spike}}/T_{\text{0}}+\tau \right].$ Then the spike generated at $t_{3,n-1}^{s\text{pike}}$ by oscillator 3 will arrive when oscillator 2 has phase $\chi_{b}\left(\varepsilon \right)+\left[\text{1}+\beta_{b}\left(\varepsilon \right)\right]\left[t_{1,n-1}^{\text{spike}}/T_{\text{0}}+\tau \right]+\left(t_{3,n-1}^{\text{spike}}-t_{1,n-1}^{\text{spike}}\right)/T_{\text{0}}$ and this causes a change in phase of oscillator 2 to

$$\begin{array}{llll} \varphi_{2}^{\text{new}} & \equiv \left[1+\beta_{b}\left(\varepsilon \right)\right]\left\{\chi_{b}\left(\varepsilon \right)+\left[\text{1}+\beta_{b}\left(\varepsilon \right)\right]\left[t_{1,n-1}^{\text{spike}}/T_{\text{0}}+\tau \right]+\left(t_{3,n-1}^{\text{spike}}-t_{1,n-1}^{\text{spike}}\right)/T_{\text{0}}\right\}+\chi_{b}\left(\varepsilon \right), & & \text{(A20)}\\ \Delta t & \equiv \left(1-\varphi_{2}^{\text{new}}\right)T_{\text{0}}, & & \text{(A21)} \end{array}$$

with oscillator 2 spiking after this additional time Δ*t* has passed. Hence, the phases of the outer oscillators at the time $t_{2,n}^{\text{spike}},$ when oscillator 2 spikes, are

$$\begin{array}{llll} {\tilde{\varphi }}_{1} & =\tau +\frac{\Delta t}{T_{\text{0}}}+\frac{t_{3,n-1}^{\text{spike}}-t_{1,n-1}^{\text{spike}}}{T_{\text{0}}}, & & \text{(A22)}\\ {\tilde{\varphi }}_{3} & =\tau +\frac{\Delta t}{T_{\text{0}}}. & & \text{(A23)} \end{array}$$

The corresponding Jacobian matrix is

(A24) $$\left\{\;\begin{array}{cc} \left[1+\beta_{b}\left(\varepsilon \right)\right]\left\{1+\beta_{b}\left(\varepsilon \right)\left[1+\beta_{b}\left(\varepsilon \right)\right]\right\} & \left[1+\beta_{b}\left(\varepsilon \right)\right]\beta_{b}\left(\varepsilon \right)\\ \beta_{b}\left(\varepsilon \right){\left[1+\beta_{b}\left(\varepsilon \right)\right]}^{2} & {\left[1+\beta_{b}\left(\varepsilon \right)\right]}^{2} \end{array}\right\},$$

with resulting eigenvalues

(A25) $$\lambda_{1}=1+\beta_{b}\left(\varepsilon \right)\text{, }\lambda_{2}={\left[1+\beta_{b}\left(\varepsilon \right)\right]}^{3}.$$

This fixed point is not stable, because these eigenvalues exceed the value one for ε∈(0, 0.21). In a similar manner one can show that Eq. A13 “NDS2” and “NDS3” in Appendix are unstable.

#### Impact of STDP on coupling in slave synchrony

In slave synchrony, the coupling strengths from the outer oscillators to the relay oscillator remain unchanged by STDP since the relay oscillator instantly spikes upon input from the outer oscillators. How about the coupling from the relay oscillator to the outer oscillators? Say their strengths are ε*_n - 1_* just after the outer oscillators spike at $t_{1,n-1}^{\text{spike}}=t_{3,n-1}^{\text{spike}}.$ The relay spike arrives when the outer oscillators have phase 2τ, since the relay oscillator fired at their phase τ. Considering all prior spikes of the outer oscillators at times $t_{1,l}^{\text{spike}},$ this new relay spike arriving at time $t_{1,n-1}^{\text{spike}}+2\tau T_{\text{0}}$ changes the coupling strength by

(A26) $${\tilde{\varepsilon }}_{n-1}=\varepsilon_{n-1}+\varepsilon_{n-1}\overset{n-1}{\underset{l=0}{\sum }}W\left[t_{1,l}^{\text{spike}}-\left(t_{1,n-1}^{\text{spike}}+2\tau T_{\text{0}}\right)\right]/60<\varepsilon_{n-1}.$$

Note that this change is a decrease in coupling strength because $t_{1,l}^{\text{spike}}<t_{1,n-1}^{\text{spike}}+2\tau T_{\text{0}}$ for *l* = 0, 1, …, *n* − 1 and therefore $W\left[t_{1,l}^{\text{spike}}-\left(t_{1,n-1}^{\text{spike}}+2\tau T_{\text{0}}\right)\right]<0.$ The input from the relay oscillator also causes a phase shift Δφ(2τ|ε*_n − 1_*) of the outer oscillators such that their next spike occurs at $t_{1,n}^{\text{spike}}=t_{1,n-1}^{\text{spike}}+T_{\text{0}}-T_{\text{0}}\Delta \varphi \left(2\tau \;\left|\;\varepsilon_{n-1}\right.\right)$. Hence the new relay spike arrival at the outer oscillators precedes the next outer oscillator spike by (1 − 2τ)*T*_0_ − *T*_0_Δφ(2τ|ε*_n - 1_*), and the previous relay spikes arrive at the outer oscillators at $t_{1,n-1}^{\text{spike}}-t_{1,k}^{\text{spike}}$ for *k* = 0, 1, …, *n* − 2 earlier than the new relay spike arrival. Therefore, all previous synaptic inputs considered as a whole lead to an increase of the synaptic weight by

(A27) $$\varepsilon_{n}={\tilde{\varepsilon }}_{n-1}+{\tilde{\varepsilon }}_{n-1}\overset{n-1}{\underset{k=0}{\sum }}W\left[\left(1-2\tau \right)T_{\text{0}}-T_{\text{0}}\Delta \varphi \left(2\tau \;\left|\;\varepsilon_{n-1}\right.\right)+t_{1,n-1}^{\text{spike}}-t_{1,k}^{\text{spike}}\right]/60>{\tilde{\varepsilon }}_{n-1}.$$

Under certain conditions, see main text, this leads to STDP turning slave synchrony into driven synchrony, and the transition time can be determined by numerically iterating the formulas for ${\tilde{\varepsilon }}_{n-1}$ and ε*_n_*.

## References

[B1] Bi G.-Q. Poo M.-M. (1998). Synaptic modifications in cultured hippocampal neurons: dependence on spike timing, synaptic strength, and postsynaptic cell type. J. Neurosci. 18, 10464–10472 9852584 10.1523/JNEUROSCI.18-24-10464.1998 PMC6793365

[B2] Börgers C. Krupa M. Gielen S. (2010). The response of a classical Hodgkin-Huxley neuron to an inhibitory input pulse. J. Comput. Neurosci. 28, 509–526 10.1007/s10827-010-0233-8 20387110 PMC2880705

[B3] Bressloff P. C. Coombes S. (1998). Desynchronization, mode locking, and bursting in strongly coupled integrate-and-fire oscillators. Phys. Rev. Lett. 81, 2168 10.1103/PhysRevLett.81.2168

[B4] Bressloff P. C. Coombes S. (2000). Dynamics of strongly coupled spiking neurons. Neural Comput. 12, 91–129 10.1162/089976600300014809 10636934

[B5] Buonomano D. V. Maass W. (2009). State-dependent computations: spatiotemporal processing in cortical networks. Nat. Rev. Neurosci. 10, 113–125 10.1038/nrn2558 19145235

[B6] Castelo-Branco M. Neuenschwander S. Singer W. (1998). Synchronization of visual responses between the cortex, lateral geniculate nucleus, and retina in the anesthetized cat. J. Neurosci. 18, 6395–6410 9698331 10.1523/JNEUROSCI.18-16-06395.1998 PMC6793201

[B7] Engel A. K. Konig P. Kreiter A. K. Schillen T. B. Singer W. (1992). Temporal coding in the visual-cortex – new vistas on integration in the nervous-system. Trends Neurosci. 15, 218–226 10.1016/0166-2236(92)90039-B 1378666

[B8] Engel A. K. Singer W. (2001). Temporal binding and the neural correlates of sensory awareness. Trends Cogn. Sci. (Regul. Ed.) 5, 16–25 10.1016/S1364-6613(00)01568-0 11164732

[B9] Erisir A. Lau D. Rudy B. Leonard C. S. (1999). Function of specific K+ channels in sustained high-frequency firing of fast-spiking neocortical interneurons. J. Neurophysiol. 82, 2476–2489 10561420 10.1152/jn.1999.82.5.2476

[B10] Ermentrout B. (1996). Type I membranes, phase resetting curves, and synchrony. Neural Comput. 8, 979–1001 10.1162/neco.1996.8.5.979 8697231

[B11] Ermentrout B. Pascal M. Gutkin B. (2001). The effects of spike frequency adaptation and negative feedback on the synchronization of neural oscillators. Neural Comput. 13, 1285–1310 10.1162/08997660152002861 11387047

[B12] Ernst U. Pawelzik K. Geisel T. (1995). Synchronization induced by temporal delays in pulse-coupled oscillators. Phys. Rev. Lett. 74, 1570–1573 10.1103/PhysRevLett.74.4416 10059062

[B13] Ernst U. Pawelzik K. Geisel T. (1998). Delay-induced multistable synchronization of biological oscillators. Phys. Rev. E 57, 2150–2162 10.1103/PhysRevE.57.2150

[B14] Fischer I. Vicente R. Buldú J. M. Peil M. Mirasso C. R. Torrent M. C. García-Ojalvo J. (2006). Zero-lag long-range synchronization via dynamical relaying. Phys. Rev. Lett. 97, 123901 10.1103/PhysRevLett.97.123902 17025966

[B15] Frien A. Eckhorn R. Bauer R. Woelbern T. Kehr H. (1994). Stimulus-specific fast oscillations at zero phase between visual areas V1 and V2 of awake monkey. Neuroreport 5, 2273–2277 10.1097/00001756-199411000-00017 7881044

[B16] Fries P. (2005). A mechanism for cognitive dynamics: neuronal communication through neuronal coherence. Trends Cogn. Sci. (Regul. Ed.) 9, 474–480 10.1016/j.tics.2005.08.011 16150631

[B17] Fries P. Reynolds J. H. Rorie A. E. Desimone R. (2001). Modulation of oscillatory neuronal synchronization by selective visual attention. Science 291, 1560–1563 10.1126/science.1055465 11222864

[B18] Fries P. Scheeringa R. Oostenveld R. (2008). Finding gamma. Neuron 58, 303–305 10.1016/j.neuron.2008.04.020 18466741

[B19] Froemke R. C. Tsay I. A. Raad M. Long J. D. Dan Y. (2006). Contribution of individual spikes in burst-induced long-term synaptic modification. J. Neurophysiol. 95, 1620–1629 10.1152/jn.00910.2005 16319206

[B20] Goel P. Ermentrout B. (2002). Synchrony, stability, and firing patterns in pulse-coupled oscillators. Physica D 163, 191–216 10.1016/S0167-2789(01)00374-8

[B21] Goldbeter A. (1996). Biochemical Oscillations and Cellular Rhythms: The Molecular Bases of Periodic and Chaotic Behaviour. Cambridge: Cambridge University Press

[B22] Gollo L. L. Mirasso C. Villa A. E. P. (2010). Dynamic control for synchronization of separated cortical areas through thalamic relay. Neuroimage 52, 947–955 10.1016/j.neuroimage.2009.11.058 19958835

[B23] Gray C. M. Konig P. Engel A. K. Singer W. (1989). Oscillatory responses in cat visual-cortex exhibit inter-columnar synchronization which reflects global stimulus properties. Nature 338, 334–337 10.1038/338680a0 2922061

[B24] Gross J. Schmitz F. Schnitzler I. Kessler K. Shapiro K. Hommel B. Schnitzler A. (2004). Modulation of long-range neural synchrony reflects temporal limitations of visual attention in humans. Proc. Natl. Acad. Sci. U.S.A. 101, 13050–13055 10.1073/pnas.0404944101 15328408 PMC516515

[B25] Hansel D. Mato G. (1993). Patterns of synchrony in a heterogeneous Hodgkin-Huxley neural network with weak coupling. Physica A 200, 662–669 10.1016/0378-4371(93)90573-M

[B26] Hansel D. Mato G. Meunier C. (1993a). Clustering and slow switching in globally coupled phase oscillators. Phys. Rev. E 48, 3470–3477 10.1103/PhysRevE.48.3470 9961005

[B27] Hansel D. Mato G. Meunier C. (1993b). Phase dynamics for weakly coupled Hodgkin-Huxley neurons. Europhys. Lett. 23, 367 10.1209/0295-5075/23/5/011

[B28] Hebb D. O. (1949). The Organization of Behaviour: A Neuropsychological Theory. New York: Wiley

[B29] Hennequin G. Gerstner W. Pfister J. P. (2010). STDP in adaptive neurons gives close-to-optimal information transmission. Front. Comput. Neurosci. 4:143 10.3389/fncom.2010.00143 21160559 PMC3001990

[B30] Hodgkin A. L. Huxley A. F. (1952). A quantitative description of membrane current and its application to conduction and excitation in nerve. J. Physiol. (Lond.) 117, 500–544 12991237 10.1113/jphysiol.1952.sp004764 PMC1392413

[B31] Izhikevich E. M. (2007). Dynamical Systems in Neuroscience: the Geometry of Excitability and Bursting. Cambridge, MA: MIT Press

[B32] Jeong H. Y. Gutkin B. (2007). Synchrony of neuronal oscillations controlled by GABAergic reversal potentials. Neural Comput. 19, 706–729 10.1162/neco.2007.19.3.706 17298230

[B33] Kawaguchi Y. (1995). Physiological subgroups of nonpyramidal cells with specific morphological-characteristics in layer Ii/Iii of rat frontal-cortex. J. Neurosci. 15, 2638–2655 7722619 10.1523/JNEUROSCI.15-04-02638.1995 PMC6577784

[B34] Knoblauch A. Sommer F. T. (2003). Synaptic plasticity, conduction delays, and inter-areal phase relations of spike activity in a model of reciprocally connected areas. Neurocomputing 52–54, 301–306. 10.1016/S0925-2312(02)00792-0

[B35] Koch C. Segev I. (1998). Methods in Neuronal Modeling: from Ions to Networks. Cambridge, MA: MIT Press

[B36] Lee S. Sen K. Kopell N. (2009). Cortical gamma rhythms modulate NMDAR-mediated spike timing dependent plasticity in a biophysical model. PLoS Comput. Biol. 5, e1000602 10.1371/journal.pcbi.1000602 20011119 PMC2782132

[B37] Lindner B. Gangloff D. Longtin A. Lewis J. E. (2009). Broadband coding with dynamic synapses. J. Neurosci. 29, 2076–2088 10.1523/JNEUROSCI.3702-08.2009 19228961 PMC6666327

[B38] Mirollo R. E. Strogatz S. H. (1990). Synchronization of pulse-coupled biological oscillators. SIAM J. Appl. Math. 50, 1645–1662 10.1137/0150008

[B39] Pesaran B. Pezaris J. S. Sahani M. Mitra P. P. Andersen R. A. (2002). Temporal structure in neuronal activity during working memory in macaque parietal cortex. Nat. Neurosci. 5, 805–811 10.1038/nn890 12134152

[B40] Pikovsky A. Rosenblum M. Kurths J. (2001). Synchronization: A Universal Concept in Nonlinear Sciences. Cambridge: Cambridge University Press

[B41] Prescott S. A. Ratté S. De Koninck Y. Sejnowski T. J. (2008). Pyramidal neurons switch from integrators in vitro to resonators under in vivo-like conditions. J. Neurophysiol. 100, 3030–3042 10.1152/jn.90634.2008 18829848 PMC2604842

[B42] Reyes A. D. Fetz E. E. (1993a). Effects of transient depolarizing potentials on the firing rate of cat neocortical neurons. J. Neurophysiol. 69, 1673–1683 8389835 10.1152/jn.1993.69.5.1673

[B43] Reyes A. D. Fetz E. E. (1993b). Two modes of interspike interval shortening by brief transient depolarizations in cat neocortical neurons. J. Neurophysiol. 69, 1661–1672 8389834 10.1152/jn.1993.69.5.1661

[B44] Rodriguez E. George N. Lachaux J. P. Martinerie J. Renault B. Varela F. J. (1999). Perception’s shadow: long-distance synchronization of human brain activity. Nature 397, 430–433 10.1038/17120 9989408

[B45] Roelfsema P. R. Engel A. K. Konig P. Singer W. (1997). Visuomotor integration is associated with zero time-lag synchronization among cortical areas. Nature 385, 157–161 10.1038/385157a0 8990118

[B46] Roxin A. Brunel N. Hansel D. (2005). Role of delays in shaping spatiotemporal dynamics of neuronal activity in large networks. Phys. Rev. Lett. 94, 1–4 10.1103/PhysRevLett.94.238103 16090506

[B47] Schoffelen J. M. Oostenveld R. Fries P. (2005). Neuronal coherence as a mechanism of effective corticospinal interaction. Science 308, 111–113 10.1126/science.1107027 15802603

[B48] Singer W. Gray C. M. (1995). Visual feature integration and the temporal correlation hypothesis. Annu. Rev. Neurosci. 18, 555–586 10.1146/annurev.ne.18.030195.003011 7605074

[B49] Song S. Miller K. D. Abbott L. F. (2000). Competitive Hebbian learning through spike-timing-dependent synaptic plasticity. Nat. Neurosci. 3, 919–926 10.1038/78829 10966623

[B50] Stiefel K. M. Gutkin B. S. Sejnowski T. J. (2008). Cholinergic neuromodulation changes phase response curve shape and type in cortical pyramidal neurons. PLoS ONE 3, e3947 10.1371/journal.pone.0003947 19079601 PMC2596483

[B51] Stiefel K. M. Gutkin B. S. Sejnowski T. J. (2009). The effects of cholinergic neuromodulation on neuronal phase-response curves of modeled cortical neurons. J. Comput. Neurosci. 26, 289–301 10.1007/s10827-008-0111-9 18784991 PMC2857973

[B52] Tateno T. Harsch A. Robinson H. P. C. (2004). Threshold firing frequency-current relationships of neurons in rat somatosensory cortex: Type 1 and type 2 dynamics. J. Neurophysiol. 92, 2283–2294 10.1152/jn.00109.2004 15381746

[B53] Theyel B. B. Llano D. A. Sherman M. (2010). The corticothalamocortical circuit drives higher-order cortex in the mouse. Nat. Neurosci. 13, 84–88 10.1038/nn.2449 19966840 PMC2846438

[B54] Tsubo Y. Takada M. Reyes A. D. Fukai T. (2007). Layer and frequency dependencies of phase response properties of pyramidal neurons in rat motor cortex. Eur. J. Neurosci. 25, 3429–3441 10.1111/j.1460-9568.2007.05579.x 17553012

[B55] van Rossum M. C. W. Bi G. Q. Turrigiano G. G. (2000). Stable Hebbian learning from spike timing-dependent plasticity. J. Neurosci. 20, 8812–8821 11102489 10.1523/JNEUROSCI.20-23-08812.2000 PMC6773092

[B56] van Vreeswijk C. Abbott L. F. Ermentrout G. B. (1994). When inhibition not excitation synchronizes neural firing. J. Comput. Neurosci. 1, 313–321 10.1007/BF00961879 8792237

[B57] Vicente R. Gollo L. L. Mirasso C. R. Fischer I. Pipa G. (2008). Dynamical relaying can yield zero time lag neuronal synchrony despite long conduction delays. Proc. Natl. Acad. Sci. U.S.A. 105, 17157–17162 10.1073/pnas.0809353105 18957544 PMC2575223

[B58] Wang S. Chandrasekaran L. Fernandez F. R. White J. A. Canavier C. C. (2012). Short conduction delays cause inhibition rather than excitation to favor synchrony in hybrid neuronal networks of the entorhinal cortex. PLoS Comput. Biol. 8, e1002306 10.1371/journal.pcbi.1002306 22241969 PMC3252263

[B59] Woodman M. M. Canavier C. C. (2011). Effects of conduction delays on the existence and stability of one to one phase locking between two pulse-coupled oscillators. J. Comput. Neurosci. 31, 401–418 10.1007/s10827-011-0315-2 21344300 PMC3130804

[B60] Zeitler M. Daffertshofer A. Gielen C. C. (2009). Asymmetry in pulse-coupled oscillators with delay. Phys. Rev. E Stat. Nonlin. Soft Matter Phys. 79, 065203 10.1103/PhysRevE.79.065203 19658549

